# Osteoblast‐Derived ECM1 Promotes Anti‐Androgen Resistance in Bone Metastatic Prostate Cancer

**DOI:** 10.1002/advs.202407662

**Published:** 2024-11-20

**Authors:** Xinwen Wang, Min Wang, Qijun Lin, Lixin He, Baolin Zhang, Xin Chen, Guanhong Chen, Hong Du, Chuandong Lang, Xinsheng Peng, Yuhu Dai

**Affiliations:** ^1^ Department of Orthopedic Surgery the First Affiliated Hospital Sun Yat‐Sen University Guangzhou 510080 China; ^2^ Guangdong Provincial Key Laboratory of Orthopedics and Traumatology Guangzhou 510080 China; ^3^ Department of Pathology Guangzhou First People's Hospital Guangzhou 510080 China; ^4^ Department of Experimental Research State Key Laboratory of Oncology in South China Collaborative Innovation Center for Cancer Medicine Sun Yat‐sen University Cancer Center Guangzhou 510060 China; ^5^ Department of Orthopedics The First Affiliated Hospital of USTC Division of Life Sciences and Medicine University of Science and Technology of China Hefei 230001 China

**Keywords:** ECM1, ENO1, enzalutamide resistance, osteoblast, prostate cancer

## Abstract

Acquired resistance to hormonal therapy, particularly enzalutamide (ENZ), remains a significant obstacle in the treatment of advanced bone metastatic prostate cancer. Here, it is demonstrated that under ENZ treatment, osteoblasts in the bone microenvironment secrete increased levels of extracellular matrix protein 1 (ECM1), which affects surrounding prostate cancer cells, promoting tumor cell proliferation and anti‐androgen resistance. Mechanistically, ECM1 interacts with the enolase 1 (ENO1) receptor on the prostate cancer cell membrane, leading to its phosphorylation at the Y189 site. This event further recruits adapter proteins including growth factor receptor‐bound protein 2 (GRB2) and son of sevenless homolog 1 (SOS1), which activates the downstream mitogen‐activated protein kinase (MAPK) signaling pathway to induce anti‐androgen resistance. Furthermore, inhibiting ECM1 or utilizing the ENO1‐targeting inhibitor phosphonoacetohydroxamate (PhAH) significantly restores tumor cell sensitivity to ENZ. Taken together, a potential mechanism is identified through which osteoblast‐derived ECM1 drives resistance in bone metastatic prostate cancer under ENZ treatment. Additionally, the findings indicate that ECM1 and ENO1 may serve as potential targets for developing therapies for bone metastatic castration‐resistant prostate cancer.

## Graphical abstract

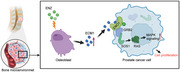

## 1 Introduction

Prostate cancer (PCa) is the second most common type of cancer among men worldwide and ranks fifth in cancer‐related mortality.^[^ ^1^ ^]^ Although the overall 5‐year survival rate for patients with early‐stage is close to 100%, the 5‐year survival rate for patients with advanced PCa is only 25%.^[^ ^2^ ^]^ The high fatality rate of PCa is primarily due to its tendency to metastasize to adjacent organs, while bone metastasis (BM) is observed in over 80% of the patients who succumb to the disease.^[^ ^3^ ^]^ Thereby, BM is the leading cause of diminished quality of life and poor prognosis in patients with PCa.

When tumor cells spread to the bones, patients often lose the opportunity for curative surgical treatment, and only palliative treatment options remain. For PCa patients with BM, hormonal therapy including androgen deprivation therapy (ADT), and anti‐androgen therapy is considered as the primary treatment approach.^[^ ^4^, ^5^ ^]^ Although hormonal therapy can achieve rapid and significant therapeutic effects in the initial stages, almost all patients eventually develop acquired resistance, leading to bone metastatic castration‐resistant PCa (bmCRPC).^[^ ^6^, ^7^ ^]^ For these patients, not only is hormonal therapy ineffective, but chemotherapy and radiation therapy also struggle to achieve satisfactory results.^[^ ^8^, ^9^ ^]^ Androgen receptor (AR) pathway inhibitor enzalutamide (ENZ) treatment can significantly alleviate disease progression in early bmCRPC patients, but resistance inevitably develops in the later stages.^[^ ^10^ ^]^ Therefore, elucidating the key molecular mechanisms underlying the development of treatment resistance in bmCRPC and designing precise therapeutic strategies is of profound importance.

The reported molecular mechanisms underlying resistance to hormonal therapy in PCa mainly include intrinsic mechanisms, such as mutations in the AR signaling pathway or the activation of bypass pathways, as well as the regulation of PCa cells by the tumor microenvironment (TME).^[^ ^11^, ^14^ ^]^ Recent single‐cell transcriptomic studies have confirmed changes in gene expression observed during ENZ treatment of metastatic prostate cancer patients, rather than selective resistance clone formation.^[^ ^15^ ^]^ This indicates that the metastatic microenvironment may induce tumor drug resistance by driving these changes in gene expression. Importantly, tumor‐stromal interactions in TME are vital for the development of resistance in PCa. Cancer‐associated fibroblasts (CAF) promote anti‐androgen resistance in PCa cells by secreting neuregulin 1 to initiate HER3 signaling.^[^ ^13^ ^]^ Furthermore, ADT‐induced secreted phosphoprotein 1 (SPP1)^+^ myofibroblastic CAFs (myCAFs) provoke the progression of castration‐resistant PCa (CRPC) by activating the extracellular regulated protein kinases (ERK) signaling pathway.^[^ ^14^ ^]^ Particularly, the interactions between the bone microenvironment and tumor cells have attracted considerable attention. In contrast to other stromal cells, osteoblasts are specific and have received less attention in bmCRPC. Sphingosine‐1‐phosphate secreted by osteoblasts promotes cell proliferation and induces therapeutic resistance in bone metastatic PCa (BMPC) cells.^[^ ^16^ ^]^ Zheng et al. also confirmed that Jagged1 originating from osteoblasts activates the Notch signaling pathway, promoting breast cancer BM that are insensitive to chemotherapy.^[^ ^17^ ^]^ However, the mechanisms by which bone metastatic prostate cancer cells develop resistance to standard hormonal therapy through interactions with osteoblasts in the bone microenvironment remain poorly understood.

Extracellular matrix protein 1 (ECM1) was initially identified as a glycoprotein secreted by mouse osteoblasts, which participated in cell proliferation, differentiation, migration, and extracellular matrix remodeling.^[^ ^18^ ^]^ Our study found that under the pressure of ENZ, osteoblasts increased the secretion of ECM1 protein, facilitating resistance to ENZ in PCa cells. Specifically, our data revealed that ECM1 can bind to enolase 1 (ENO1), which acts as a receptor on the membrane of PCa cells, inducing its phosphorylation. This further recruits adapter proteins growth factor receptor‐bound protein 2 (GRB2) and son of sevenless homolog 1 (SOS1), activating the downstream mitogen‐activated protein kinase (MAPK) signaling pathway to promote ENZ resistance. Furthermore, inhibiting ECM1 or using inhibitors specifically targeting ENO1 significantly suppressed the growth of therapy‐resistant PCa cells in the bone. Taken together, our findings reveal an innovative mechanism by which osteoblasts‐derived ECM1 contributes to anti‐androgen resistance in bmCRPC and provide potential therapeutic strategies.

## 2 Results

### 2.1 Osteoblasts in the Bone Microenvironment Promote the Resistance of PCa Cells to ENZ

**Figure 1:**
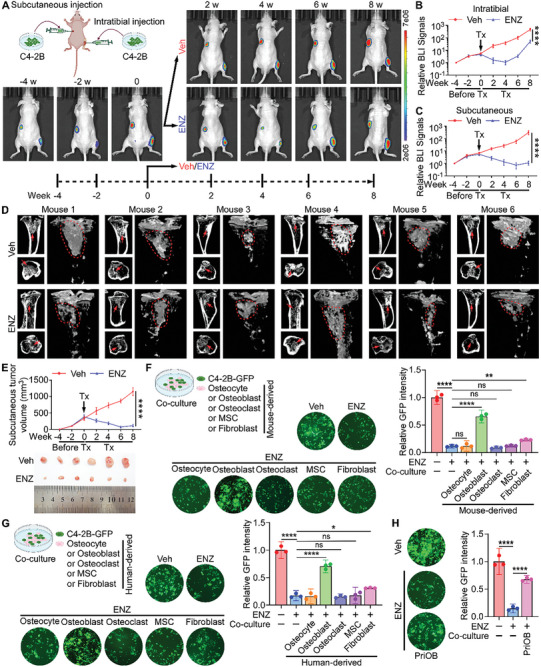
Osteoblasts in the bone microenvironment promote the resistance of PCa cells to ENZ. A) Representative BLI of subcutaneous and intratibial tumors treated daily with vehicle (Veh, n = 6) or enzalutamide (ENZ, 20 mg kg^−1^, n = 6) by oral gavage at 4 weeks and 2 weeks before treatment, and at weeks 0, 2, 4, 6, and 8 during treatment, as shown in the treatment schematic diagram below. B,C) Quantification of BLI signals in intratibial and subcutaneous tumors of mice before and after treatment (Tx) as indicated in A (n = 6 per group). D) Representative micro‐CT images of intratibial lesions from mice after 8 weeks of treatment (arrows and circles indicate osteoblastic lesions, n = 6 per group). E) Growth curves (top) and representative images (bottom) of subcutaneous tumors in mice as indicated in A. Tumor volumes were measured weekly (n = 6 per group). F,G) Schematic diagram (top left) of C4‐2B‐GFP cells (green) co‐cultured with several murine‐derived cells (F) or human‐derived cells (G). Representative fluorescent images showing C4‐2B‐GFP cells cultured alone or co‐cultured with several murine‐derived cells or human‐derived cells in the presence of ENZ (10 µM) on day 7, compared to untreated cells (bottom left). Quantification of GFP fluorescence intensity from the fluorescent images on the left (right, n = 3 per group). H) Representative fluorescent images showing C4‐2B‐GFP cells cultured alone or co‐cultured with mouse‐derived primary osteoblasts (PriOBs) with the addition of ENZ (10 µM) on day 7, compared to untreated cells (left). Quantification of GFP fluorescence intensity from the fluorescent images on the left (right, n = 3 per group). ns, not significant; *, *P* < 0.05; **, *P* < 0.01; ***, *P* < 0.001; ****, *P* < 0.0001.

To investigate the role of the bone microenvironment in bone metastatic PCa, we established a mouse tumor model. GFP‐luciferase‐labeled C4‐2B PCa cells were injected into the subcutaneous tissue and tibia of 4‐week‐old BALB/c‐nu mice. That is an AR‐positive PCa bone metastatic cell line that is androgen‐independent, making it an ideal cell line for bmCRPC modeling.^[^ ^19^ ^]^ After 4 weeks, ENZ (20 mg kg^−1^) or vehicle was administered daily through oral gavage for 8 consecutive weeks. The mice were monitored every three days for BM using bioluminescence imaging (BLI) and subcutaneous tumor volumes were measured weekly (**Figure** **1**A). Compared to the vehicle group, ENZ treatment significantly inhibited the growth of C4‐2B subcutaneous tumors within six weeks, but only decreased the fluorescence signal of intratibial tumors during the initial 4 weeks, with tumors continuing to grow thereafter (Figure 1B,C). Mice were sacrificed after eight weeks of drug administration, and both subcutaneous and intratibial tumors were harvested for measurement. Micro‐CT scans of the tibiae and quantitative analysis of bone parameters, including the bone/tissue volume ratio (BV/TV), bone surface/tissue volume ratio (BS/TV), relative trabecular number (Tb.n), and trabecular thickness (Tb.th), suggested no significant differences between the two groups (Figure 1D; Figure S1A, Supporting Information). Since C4‐2B is an osteoblastic and osteolytic mixed cell line, the degree of bone destruction in the mouse tibiae does not fully reflect the size of the tumor. However, subcutaneous tumor volumes in the ENZ group were significantly smaller than those in the vehicle group (Figure 1E). Thus, we speculated that differences in the microenvironment in which tumor cells reside may cause variability in their sensitivity to ENZ. To verify this hypothesis, we isolated ENZ‐resistant intratibial tumor tissue, sorted out tumor cells via GFP labeling, re‐inoculated them into subcutaneous tissue and tibiae (Figure S1B, Supporting Information), and found that tumors growing in the tibiae remained resistant to ENZ (Figure S1C,D, Supporting Information), whereas those growing subcutaneously exhibited sensitivity to ENZ (Figure S1E, Supporting Information). Based on these observations, we hypothesized that ENZ resistance does not originate inherently from PCa cells, but is likely induced by the presence of the bone microenvironment, which may lead to the development of ENZ resistance in patients with BM.

The bone microenvironment is a unique and complex area, characterized not only by abundant fibroblasts, mesenchymal stem cells (MSCs), and immune cells common to other microenvironments,^[^ ^13^, ^20^, ^21^ ^]^ but also by a large number of osteocytes, osteoblasts, and osteoclasts.^[^ ^22^ ^]^ To explore the impact of diverse stromal cells in the bone microenvironment on PCa cells, we constructed a co‐culture model composed of C4‐2B‐GFP cells and mouse‐derived osteocytes, osteoblasts, osteoclasts, MSCs, or fibroblasts, cultured in the presence of ENZ. The GFP fluorescence intensity was measured to assess the relative number of C4‐2B cells. Our findings demonstrated that ENZ‐treated osteoblasts significantly promoted C4‐2B cell proliferation, far exceeding the effects of the other four cell types (Figure 1F). Moreover, similar phenomena were also observed in the co‐culture system of human‐derived cells and C4‐2B cells as seen with the mouse‐derived cells (Figure 1G). Additionally, mouse primary osteoblasts (PriOBs) showed enhanced proliferation of C4‐2B cells following ENZ treatment (Figure 1H). Collectively, these results indicate that under ENZ pressure, osteoblasts in the bone microenvironment promote ENZ resistance in PCa cells.

### 2.2 Osteoblast‐Derived ECM1 Induces PCa Cell Resistance to ENZ

**Figure 2:**
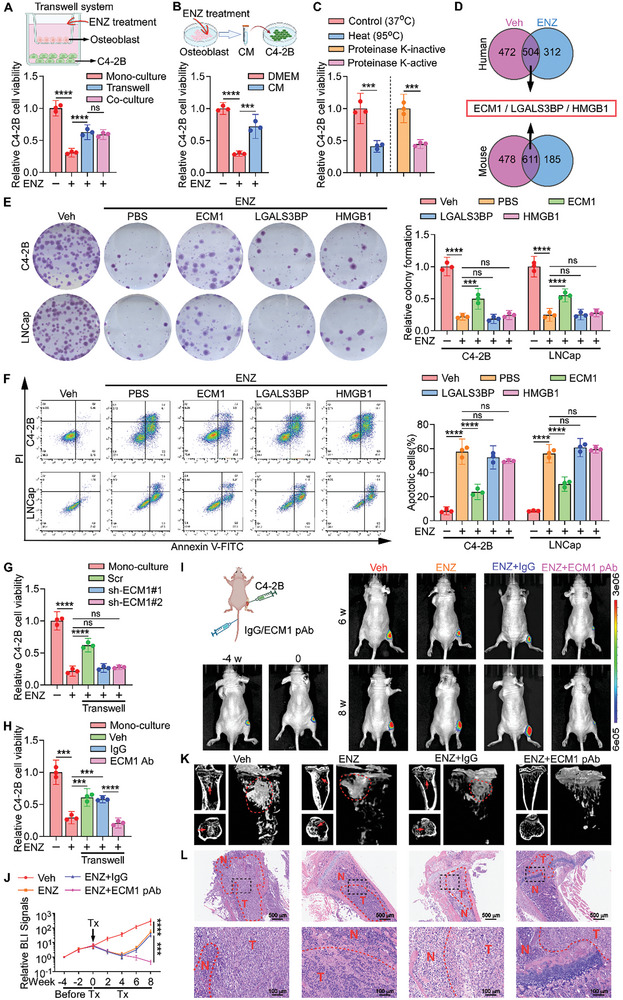
Osteoblast‐derived ECM1 induces PCa cell resistance to ENZ. A) Transwell system model (top) of C4‐2B cells with mouse‐derived osteoblasts (MC3T3‐E1), and cell proliferation of C4‐2B cells in Transwell system, cultured alone or co‐cultured with osteoblasts in the presence of ENZ (10 µM) on day 7, compared to untreated cells (bottom). B) Schematic diagram of CM from mouse‐derived osteoblasts treated with ENZ for 72 hours added to C4‐2B cells (top); Cell proliferation of C4‐2B cells cultured alone or with CM from ENZ‐treated osteoblasts in the presence of ENZ (10 µM) on day 7, compared to untreated cells (bottom). Medium Control: DMEM. C) Cell proliferation of C4‐2B cells on day 7 supplemented with either control or heat‐inactivated CM (left), and with the addition of CM treated with either proteinase K (200 µg mL^−1^) or inactivated proteinase K (right). D) Schematic diagram showing the identification of highly abundant, secreted proteins ECM1, LGALS3BP, and HMGB1 from the supernatant of human or mouse osteoblasts treated with ENZ (10 µM) or Veh (DMSO) for 72 h, followed by LC‐MS/MS analysis. E) Representative images (left) and quantification (right) of surviving colonies formed by C4‐2B and LNCaP cells treated with ENZ (10 µM) with the addition of either PBS, ECM1 (200 ng mL^−1^), LGALS3BP (200 ng mL^−1^), or HMGB1 (200 ng mL^−1^) protein, compared to untreated cells. F) Flow cytometry analysis showing representative images (left) and quantification (right) of apoptosis in C4‐2B and LNCaP cells treated as grouped in E. G) Cell proliferation on day 7 of either the indicated C4‐2B cells cultured with osteoblasts in the Transwell system, or C4‐2B cells cultured alone, in the presence of ENZ (10 µM), compared to untreated cells. H) Cell proliferation on day 7 of C4‐2B cells cultured alone or cultured with osteoblasts in the Transwell system in the presence of ENZ (10 µM), with the addition of either IgG or ECM1 antibody, compared to untreated cells. I) Representative BLI of intratibial tumors in mice treated daily with oral Veh, ENZ (20 mg kg^−1^), ENZ (20 mg kg^−1^) combined with tail vein injection of either IgG or ECM1 pAb (50 mg kg^−1^) twice weekly at 4 weeks before treatment, and at weeks 0, 6, and 8 during treatment (n = 6 per group). J) Quantification of BLI signals in intratibial tumors of mice before and after Tx as grouped in I (n = 6 per group). K) Representative micro‐CT images of intratibial lesions from mice after 8 weeks of treatment grouped as shown in I (n = 6 per group). L) Representative H&E images of intratibial tumors grouped as shown in I (T, tumor; N, the adjacent non‐tumor tissues. Scale bars, 500 µm and 100 µm). ns, not significant; *, *P* < 0.05; **, *P* < 0.01; ***, *P* < 0.001; ****, *P* < 0.0001.

Subsequently, we investigated how osteoblasts promote the resistance of PCa cells to ENZ. To exclude the effects of direct cell‐cell contact, PCa cells were cultured with mouse‐derived osteoblasts in a Transwell plate. Upon addition of ENZ, the number of PCa cells in the Transwell system was significantly higher than that when cultured alone, whereas there was no substantial difference compared to the osteoblast‐PCa co‐culture group (**Figure** **2**A). We further hypothesized that the promotion of PCa cell growth by osteoblasts during ENZ treatment is mediated by the secretion of soluble factors. We then collected conditioned medium (CM) from mouse‐derived osteoblasts treated with ENZ for 72 h to test their ability to stimulate PCa cell growth and found that the number of C4‐2B cells significantly increased in the CM group (Figure 2B). Moreover, exposure of CM to heat (95 °C) or proteinase K attenuated its ability to restore the PCa cell viability (Figure 2C), suggesting that the soluble factors promoting resistance were proteins. To pinpoint the key protein(s) responsible for ENZ resistance in the CM of ENZ‐treated osteoblasts, we conducted Liquid chromatography/tandem mass spectrometry (LC‐MS/MS) analysis of the culture supernatants from two groups of osteoblasts derived from mice and humans. The intersection of differentially secreted proteins from ENZ‐treated osteoblasts of both origins was analyzed (Figure 2D), ECM1, Galectin 3 Binding Protein (LGALS3BP), and High Mobility Group Box 1 (HMGB1) were selected based on their higher abundance, exocrine type, and good interspecies conservation characteristics. To verify whether these three secretory proteins contribute to ENZ resistance in PCa, we introduced another PCa cell line, LNCaP, which is androgen‐dependent.^[^ ^14^ ^]^ Interestingly, compared to the other groups, ECM1 significantly promoted the growth of PCa cells in the presence of ENZ addition (Figure 2E; Figure S2A, Supporting Information). Additionally, ECM1 rescued ENZ‐induced apoptosis in C4‐2B and LNCap cells (Figure 2F). Consistently, ECM1 reduced PCa cell apoptosis caused by docetaxel compared to that in the vehicle group (Figure S2B, Supporting Information), and rescued the inhibitory effect of docetaxel on the colony formation capability of PCa cells (Figure S2C, Supporting Information). Increasing the concentration of ECM1 or concentrating the CM of ENZ‐treated osteoblasts significantly enhanced the proliferation‐promoting activity by 2‐ to 4‐fold, indicating a dose‐dependent effect (Figure S2D,E, Supporting Information). In contrast, we found that the significant proliferation of PCa cells in the ENZ‐treated Transwell group could be reversed by knocking out the ECM1 gene in osteoblasts (Figure 2G) or by adding ECM1 antibodies (Figure 2H).

Furthermore, we utilized Enzyme‐linked immunosorbent assay (ELISA) to detect the levels of ECM1 in the supernatants of ENZ‐treated mouse osteocytes, osteoblasts, osteoclasts, MSCs, and fibroblasts. The findings showed that osteoblasts significantly upregulated ECM1 production in response to ENZ treatment, and fibroblasts showed a slight upregulation, while other cell types showed no noticeable differences between the two groups (Figure S2F, Supporting Information). Moreover, with increasing concentrations of ENZ treatment, a progressive elevation in both ECM1 mRNA and protein expression in osteoblasts was observed (Figure S2G,H, Supporting Information). In addition, AR was reported as a globally acting transcriptional repressor.^[^ ^23^, ^24^ ^]^ Therefore, we sought to determine whether ECM1 is transcriptionally regulated by AR. A luciferase reporter assay demonstrated that AR reduced the activity of the ECM1 promoter in both MC3T3‐E1 and hFOB1.19 cells after dihydrotestosterone (DHT) treatment (Figure S2I, Supporting Information). A chromatin immunoprecipitation (ChIP) assay also confirmed the binding of AR to the ECM1 promoter region at the presence of DHT (Figure S2J, Supporting Information). These results reveal that AR inhibits ECM1 expression at the transcriptional level.

Additionally, a BALB/c‐nu mouse BM tumor model was established by injecting C4‐2B‐luciferase cells into the right tibia. Four weeks later, ENZ was administered orally, and either IgG or ECM1 polyclonal antibody (pAb) was injected via the tail vein (Figure 2I). The BLI results showed that the progression of intratibial tumors in mice was significantly halted after injection of the ECM1 antibody (Figure 2J). Furthermore, micro‐CT scanning (Figure 2K) and Hematoxylin‐eosin (H&E) staining (Figure 2L) of mouse tibiae harvested in the 8th week suggested that the ECM1 pAb delayed tumor progression and reduced tumor lesions volume (Figure S2K, Supporting Information). Similarly, C4‐2B cells isolated from mouse intratibial tumors were implanted subcutaneously into new BALB/c‐nu mice, and then injected subcutaneously with either phosphate‐buffered saline (PBS) or ECM1 protein. After 8 weeks, the volume of subcutaneous tumors in the ECM1 group was significantly larger than that in the PBS group (Figure S2L, Supporting Information). Taken together, these results demonstrate that osteoblast‐derived ECM1 exhibits as a key factor in mediating PCa cell resistance to ENZ.

### 2.3 ECM1 Activates the MAPK Signaling Pathway in PCa Cells

**Figure 3:**
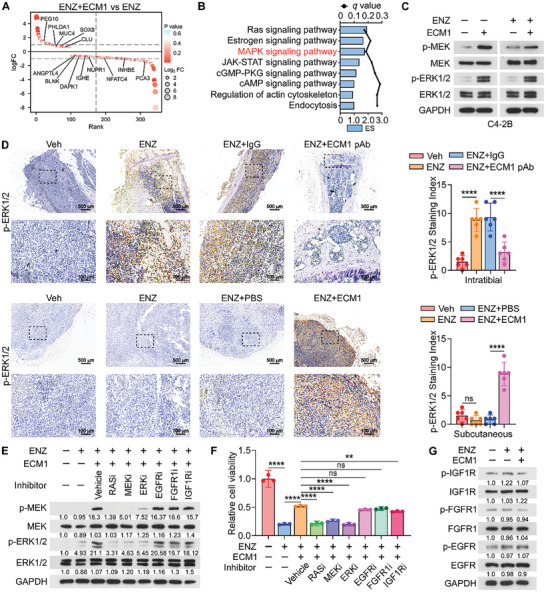
ECM1 activates the MAPK signaling pathway in PCa cells. A) Waterfall plot showing differentially expressed genes (p < 0.05, log2 fold change>1.5) in C4‐2B cells treated with ENZ (10 µM, 48 h) combined with ECM1 protein (200 ng mL^−1^, 48 h) or ENZ (10 µM, 48 h) alone. The highlighted genes were related to proliferation and apoptosis. B) KEGG analysis of pathways enriched in ENZ combined with ECM1 protein treatment group compared to the ENZ treatment group. C) WB analysis of MEK, p‐MEK, ERK1/2, and p‐ERK1/2 expression in the indicated groups of C4‐2B cells. D) IHC staining and quantification of p‐ERK1/2 expression in mice intratibial and subcutaneous tumors grouped as indicated (Scale bars, 500 µm and 100 µm, n = 6 per group). E) WB analysis and quantification of MEK, p‐MEK, ERK1/2, and p‐ERK1/2 expression in C4‐2B cells stimulated with ENZ (10 µM) combined with ECM1 (200 ng mL^−1^) in the presence of inhibitors for either RAS (MCP110, 10 µM), MEK (U0126, 10 µM), ERK1/2 (Ulixertinib, 10 µM), EGFR (Lapatinib, 10 µM), FGFR1 (Fexagratinib, 10 µM), IGF1R (Linsitinib, 10 µM) or Veh (DMSO), compared to ENZ‐treated alone or untreated C4‐2B cells. F) C4‐2B cell proliferation on day 7 of groups as shown in E. G) WB analysis and quantification of EGFR, p‐EGFR, FGFR1, p‐FGFR1, IGF1R and p‐IGF1R expression in groups as indicated. ns, not significant; *, *P* < 0.05; **, *P* < 0.01; ***, *P* < 0.001; ****, *P* < 0.0001.

To clarify the mechanism by which ECM1 promoted ENZ resistance in PCa cells, we conducted a whole‐transcriptome analysis of C4‐2B cells treated with ENZ, recombinant ECM1, or both ENZ and ECM1 simultaneously (p < 0.05, log2 fold change >1.5). ECM1 upregulated genes related to proliferation and downregulated genes associated with apoptosis (**Figure** **3**A; Figure S3A, Supporting Information), both in the presence and absence of ENZ. Furthermore, Kyoto Encyclopedia of Genes and Genomes (KEGG) pathway analysis revealed that in the ECM1‐treated group with addition of ENZ, multiple functional pathways were found to be regulated, with a remarkable enrichment of the MAPK signaling pathway (Figure 3B), which was further validated by western blotting for mitogen‐activated protein kinase kinase 1 (MEK), ERK1/2, and their phosphorylation levels (Figure 3C; Figure S3B, Supporting Information). Numerous studies have reported that activation of the MAPK signaling pathway promotes castration resistance and tumor progression in PCa.^[^ ^25^, ^26^, ^27^, ^28^ ^]^ Additionally, immunohistochemistry (IHC) staining suggested that in subcutaneous tumors, the presence of ECM1 activated the MAPK signaling pathway; similarly, in ENZ‐treated tibial tumors, the MAPK signaling pathway was also activated, whereas ECM1 pAb inhibited this process (Figure 3D).

AR signaling is crucial for the development of PCa, and reactivation of the AR signaling pathway in BMPC is one of the main mechanisms contributing to acquired resistance to AR‐targeted therapies.^[^ ^29^, ^30^ ^]^ Thus, we investigated whether ECM1 could reactivate the AR signaling pathway in tumor cells even in the presence of ENZ. The results indicated that several canonical AR target genes were suppressed by ENZ but could not be rescued by ECM1 (Figure S3C, Supporting Information). Similar results were observed in intratibial and subcutaneous tumors in mice (Figure S3D,E, Supporting Information), suggesting that ECM1 may promote tumor resistance by activating AR bypass pathways rather than by reactivating the AR itself. Gene Set Enrichment Analysis (GSEA) results also indicated that the AR signaling pathway was not significantly enriched in the ECM1‐treated group (Figure S3F, Supporting Information).

Given that the MAPK signaling pathway is primarily activated by the classical receptor tyrosine kinases (RTKs) family in various tumors,^[^ ^31^, ^32^, ^33^ ^]^ we explored whether the activation of the MAPK pathway by ECM1 was dependent on RTKs by adding small‐molecule inhibitors to RTKs. The results demonstrated that MAPK signaling pathway activation induced by ECM1 was significantly suppressed by MAPK inhibitors, but not by inhibitors of epidermal growth factor receptor (EGFR), fibroblast growth factor receptor 1 (FGFR1), or insulin like growth factor 1 receptor (IGF1R) (Figure 3E). Similarly, the proliferation‐promoting effect of ECM1 on tumor cells was not blocked by RTK inhibitors (Figure 3F). Moreover, ECM1 did not induce RTKs activation (Figure 3G). These findings suggest that ECM1 activates the MAPK signaling pathway and promotes the proliferation of PCa cells, independent of AR and RTKs activation.

### 2.4 ECM1 Recruits GRB2 and SOS1 to the Membrane to Activate the MAPK Signaling Pathway

**Figure 4:**
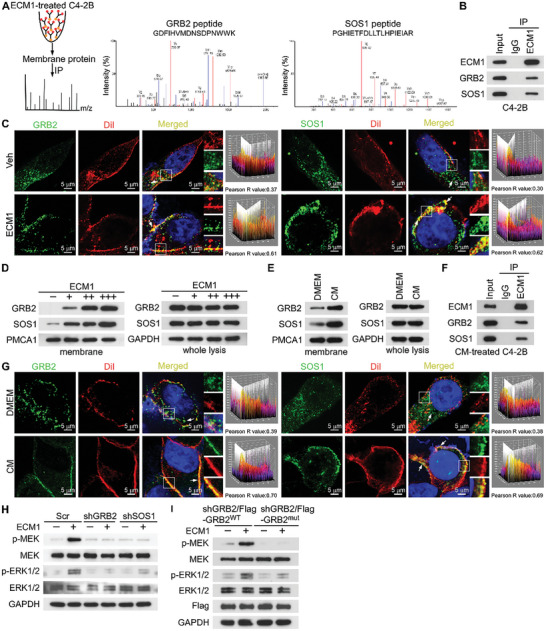
ECM1 recruits GRB2 and SOS1 to the membrane to activate the MAPK signaling pathway. A) Affinity purification MS analysis of ECM1 interaction complexes in C4‐2B cells (left). Representative mass spectra of GRB2 and SOS1 peptides (right). B) IP analysis detected the interaction between ECM1 and GRB2 as well as SOS1 in C4‐2B cells with ECM1 (200 ng mL^−1^) treatment. C) IF staining and quantification of GRB2, SOS1, and DiI in C4‐2B cells treated with Veh (PBS) or ECM1 (200 ng mL^−1^). Pearson R value greater than 0.5 indicated co‐localization of the two proteins (Scale bar, 5 µm). D,E) WB analysis of GRB2 and SOS1 protein expression in whole lysis (WL) and membrane proteins from C4‐2B cells treated with increasing concentrations of ECM1 (0, 200, 400, 800 ng mL^−1^), or with the addition of either DMEM or CM. GAPDH was used as a loading control for whole lysis, and PMCA1 for membrane proteins. F) IP detection of the interaction between ECM1 and GRB2 as well as SOS1 in C4‐2B cells treated with CM. G) IF staining and quantification of GRB2, SOS1, and DiI in C4‐2B cells treated with DMEM or CM. Pearson R value greater than 0.5 indicated co‐localization of the two proteins (Scale bar, 5 µm). H,I) WB analysis of MEK, p‐MEK, ERK1/2, and p‐ERK1/2 expression in the indicated C4‐2B cells treated with or without ECM1 (200 ng mL^−1^). ns, not significant; *, *P* < 0.05; **, *P* < 0.01; ***, *P* < 0.001; ****, *P* < 0.0001.

To investigate the mechanism by which ECM1 activates the MAPK signaling pathway to promote ENZ resistance in PCa cells, membrane protein samples obtained from C4‐2B cells treated with ECM1‐Flag protein through immunoprecipitation (IP) assays were subjected to MS analysis to identify ECM1 binding proteins. Interestingly, we observed that ECM1 potentially binds to GRB2 and SOS1 (**Figure** **4**A), which could act as adapter proteins to activate the downstream MAPK signaling pathway.^[^ ^34^, ^35^, ^36^ ^]^ Co‐immunoprecipitation (co‐IP) assays conducted on ECM1‐Flag protein‐treated C4‐2B cells confirmed endogenous interactions between ECM1 and either GRB2 or SOS1 (Figure 4B), consistent with the MS findings. Moreover, immunofluorescence (IF) staining assays showed that ECM1 treatment led to the translocation of GRB2 and SOS1 from the cytoplasm to the cell membrane, and increased their co‐localization with 1,1'‐Dioctadecyl‐3,3,3',3'‐Tetramethylindocarbocyanine Perchlorate (DiI) on the membrane (Figure 4C). Strikingly, ECM1 treatment increased the levels of GRB2 and SOS1 in the cell membrane lysates, with no significant change in overall expression (Figure 4D), which was consistent with the IF assays. Similar results were observed in C4‐2B cells when CM from ENZ‐treated osteoblasts were added (Figure 4E–G). Furthermore, silencing of either GRB2 or SOS1 blocked the activation of the MAPK signaling pathway induced by ECM1 (Figure 4H). Additionally, the SH2 domain involves the binding of GRB2 to receptor proteins, thereby recruiting GRB2 and SOS1 to the membrane and mediating signal transduction.^[^ ^35^ ^]^ We stably knocking down GRB2 with transfecting PCa cells with a GRB2 mutant plasmid deficient in bases expressing the SH2 domain sequence (GRB2^mut^), and found that activation of the MAPK signaling pathway was abolished (Figure 4I). Therefore, these results reveal that ECM1 promotes the recruitment of GRB2 and SOS1 to the membrane to activate the MAPK signaling pathway.

### 2.5 Phosphorylated ENO1 Bridges ECM1 with GRB2 and SOS1 at the Membrane

**Figure 5:**
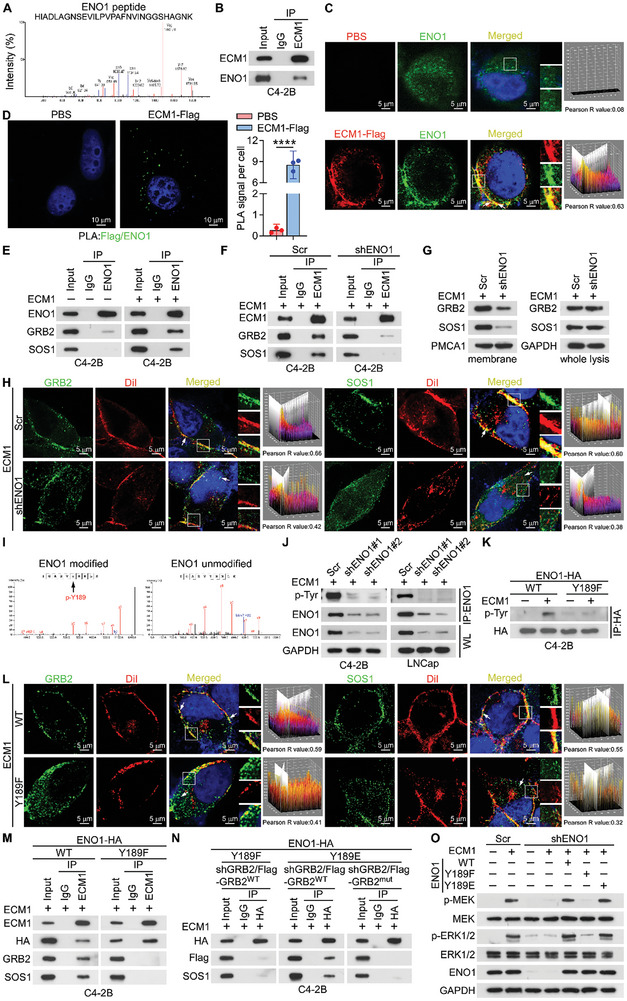
Phosphorylated ENO1 bridges ECM1 with GRB2 and SOS1 at the membrane. A) Representative mass spectra of ENO1 peptide. B) IP analysis detected the interaction between ECM1 and ENO1 in C4‐2B cells. C) IF staining and quantification of ENO1 and ECM1 in C4‐2B cells. Pearson R value greater than 0.5 indicated co‐localization of the two proteins (Scale bar, 5 µm). D) Proximity ligation assay (PLA) analysis of the interaction between ECM1‐Flag and ENO1 (Scale bar, 10 µm). E) IP assays of the interaction between ENO1 and GRB2 as well as SOS1 in C4‐2B cells in the presence or absence of ECM1 (200 ng mL^−1^). F) IP analysis detected the interaction between ECM1 and GRB2 as well as SOS1 in the indicated C4‐2B cells treated with ECM1 (200 ng mL^−1^). G) Expression of GRB2 and SOS1 protein in whole lysis and membrane proteins from the indicated C4‐2B cells with ECM1 (200 ng mL^−1^) treatment by WB analysis. H) IF staining and quantification of GRB2, SOS1, and DiI in the indicated C4‐2B cells supplemented with ECM1 (200 ng mL^−1^). Pearson R value greater than 0.5 indicated co‐localization of the two proteins (Scale bar, 5 µm). I) Representative peptides showing phosphorylation of ENO1 at the Y189 site by affinity purification MS analysis of protein modifications in ECM1‐treated C4‐2B cells. J) Tyr phosphorylation detection of the immunoprecipitated ENO1 in indicated C4‐2B and LNCap cells with ECM1 treatment (200 ng mL^−1^). K) Tyr phosphorylation levels of HA‐ENO1‐WT and ‐Y189F in C4‐2B cells with or without ECM1 (200 ng mL^−1^) treatment. L) IF staining and quantification of GRB2, SOS1, and DiI in the indicated C4‐2B cells supplemented with ECM1 (200 ng mL^−1^). Pearson R value greater than 0.5 indicated co‐localization of the two proteins (Scale bar, 5 µm). M,N) IP assays of the interaction between ECM1 and ENO1, GRB2 as well as SOS1 in the indicated C4‐2B cells in the presence of ECM1 (200 ng mL^−1^). O) WB analysis of MEK, p‐MEK, ERK1/2, and p‐ERK1/2 expression in the indicated C4‐2B cells treated with or without ECM1 (200 ng mL^−1^). ns, not significant; *, *P* < 0.05; **, *P* < 0.01; ***, *P* < 0.001; ****, *P* < 0.0001.

Since GRB2 and SOS1 are primarily located in the cytoplasm, we speculated that ECM1 recruits them to the cell membrane by binding to membrane receptors, forming a multi‐protein complex that activates downstream signaling pathway. The BioGRID database (https://thebiogrid.org) was utilized to identify binding proteins of GRB2.^[^ ^37^ ^]^ Consequently, the ECM1 interacting proteins discovered by MS analysis intersected with the binding proteins of GRB2, and we focused on ENO1 (**Figure** **5**A; Figure S4A, Supporting Information), which is localized on the cell surface and functions as a receptor.^[^ ^38^, ^39^, ^40^, ^41^ ^]^ We detected endogenous binding between ECM1 and ENO1 in ECM1‐Flag protein‐treated C4‐2B cells (Figure 5B). Simultaneously, IF analysis confirmed the co‐localization of ECM1 and ENO1 on the cell membrane (Figure 5C). A proximity ligation assay (PLA) in C4‐2B cells further confirmed ECM1‐Flag protein treatment induced a direct interaction between ECM1 and ENO1 (Figure 5D). Considering that ENO1 also functions as a glycolytic enzyme, we investigated whether ECM1 binding to ENO1 affects the glycolytic process in PCa cells. The results showed that increasing ECM1 concentration failed to affect glucose consumption and lactate production in both C4‐2B and LNCap cells (Figure S4B, Supporting Information). Furthermore, the co‐IP assays indicated that ENO1 could bind to GRB2 and SOS1 following the addition of ECM1 (Figure 5E). However, ENO1 depletion eliminated the binding of ECM1 to GRB2 and SOS1 (Figure 5F), and inhibited their recruitment to the cell membrane (Figure 5G,H). Moreover, the silencing of GRB2 removed the binding of ECM1 to SOS1 (Figure S4C, Supporting Information). Collectively, these findings reveal that the interactions of ECM1 with GRB2 and SOS1 were mediated by ENO1 (Figure S4D, Supporting Information).

We further identified the binding region between ECM1 and ENO1. ENO1 consists of three structural domains: the N‐terminal domain (NTD), catalytic domain (CTD), and plasminogen binding domain (PBD).^[^ ^38^ ^]^ ECM1 contains four structural domains: AD1, SASDL2 (S2), SASDL3 (S3), and SASDL4 (S4).^[^ ^42^ ^]^ We constructed truncated variants of ECM1 and ENO1 with different structural domains. The co‐IP results revealed that the S2 structural domain of ECM1 was bound to the PBD of ENO1 (Figure S4E, Supporting Information). Notably, ClusPro 2.0 (https://cluspro.org) was used for molecular‐docking simulations between ECM1 and ENO1.^[^ ^43^ ^]^ The 3D structure predicted from the docking results indicated that the binding region was consistent with the co‐IP results (Figure S4F, Supporting Information).

GRB2 consists of three successive Src homology (SH) domains: the N‐terminal SH3 (N‐SH3), SH2, and C‐terminal SH3 (C‐SH3).^[^ ^44^ ^]^ The SH2 domain specifically binds with high affinity to the phosphorylated tyrosine (pY) groups of receptor proteins, particularly the pYxNx motif (where x represents any natural amino acid), releasing its inhibitory state, this allows the N‐SH3 or C‐SH3 domain to interact with SOS1, activating downstream signaling pathways.^[^ ^34^, ^36^, ^44^ ^]^ Thus, we evaluated whether ENO1 undergoes phosphorylation following its interaction with ECM1, and identified the corresponding phosphorylation sites. MS analysis of the phosphorylated peptides of the endogenous ENO1 protein in ECM1‐treated C4‐2B cells indicated that Y189 was phosphorylated, while no phosphorylation was observed at any other tyrosine site (Figure 5I). Moreover, we detected ENO1 phosphorylation in immunoprecipitated ENO1 proteins using a tyrosine phosphorylation‐specific antibody (Figure 5J).

Subsequently, the Y189 site was mutated to phenylalanine (F) to mimic the dephosphorylated state.^[^ ^45^, ^46^ ^]^ Interestingly, the IP results showed that ECM1 treatment induced the phosphorylation of ENO1‐WT (wild type) but not ENO1‐Y189F (Figure 5K; Figure S4G, Supporting Information). The ENO1‐Y189F mutation blocked the binding of ECM1 to GRB2, and SOS1, and suppressed the recruitment of GRB2 and SOS1 to the membrane (Figure 5L,M; Figure S4H, Supporting Information). In addition, the ENO1‐Y189E mutant plasmid, in which the Y189 site was mutated to glutamicacid (E), was constructed to mimick the phosphorylated‐Y189 form of ENO1.^[^ ^47^, ^48^ ^]^ Notably, the expression of ENO1‐Y189E restored the binding of ENO1 to GRB2 and SOS1, which was reversed by stably knocking down GRB2 and transfecting PCa cells with a GRB2^mut^ plasmid (Figure 5N; Figure S4I, Supporting Information). Furthermore, the truncated ENO1 and GRB2 plasmids were constructed. The co‐IP results suggested that the CTD of ENO1 is bound to the SH2 domain of GRB2 (Figure S4J, Supporting Information), which was also shown in the predicted 3D docking model of the two molecules (Figure S4K, Supporting Information).

Additionally, we stably knocked out endogenous ENO1 in C4‐2B cells and re‐expressed ENO1‐WT and different ENO1 mutants. The re‐expression of ENO1‐Y189F failed to activate the MAPK signaling pathway, even with ECM1 treatment. In contrast, the re‐expression of ENO1‐Y189E activated the MAPK signaling pathway, and ENO1‐WT had a similar effect (Figure 5O). These results reveal that phosphorylation of Y189 is essential for the activation of the MAPK signaling pathway mediated by ENO1 in PCa cells.

Taken together, our results demonstrate that ECM1 binds to ENO1 on the membrane of PCa cells, and phosphorylated ENO1 links GRB2 and SOS1, further activating the downstream MAPK signaling pathway.

### 2.6 The Phosphorylation of ENO1 is Indispensable for ENZ Resistance

**Figure 6:**
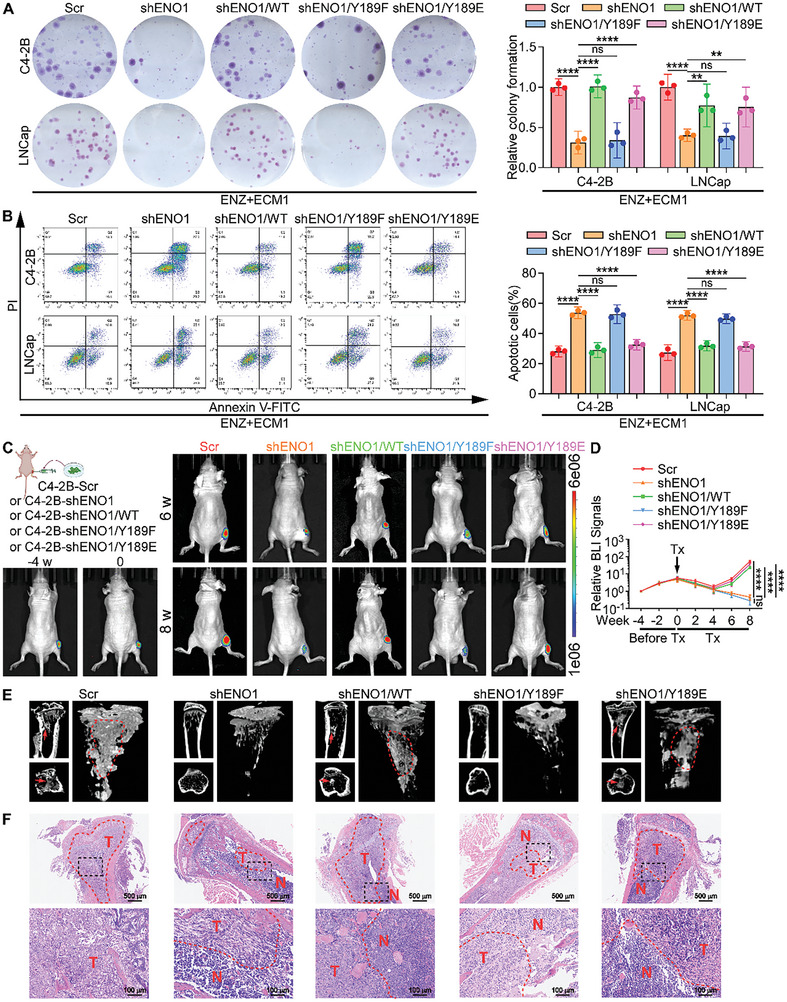
The phosphorylation of ENO1 is indispensable for ENZ resistance. A) Representative images (left) and quantification (right) of surviving colonies formed by the indicated C4‐2B and LNCaP cells treated with ENZ (10 µM) and ECM1 (200 ng mL^−1^). B) Flow cytometry analysis showing representative images (left) and quantification (right) of apoptosis in indicated C4‐2B and LNCaP cells after ENZ (10 µM) and ECM1 (200 ng mL^−1^) treatment. C,D) Representative BLI and quantification of intratibial tumors formed by the indicated C4‐2B cells in mice treated daily oral treatment with ENZ (20 mg kg^−1^) at 4 weeks before treatment, and at weeks 0, 6, and 8 during treatment (n = 6 per group). E) Representative micro‐CT images of intratibial lesions from mice after 8 weeks of treatment grouped as shown in C (arrows and circles indicate osteoblastic lesions, n = 6 per group). F) Representative H&E images of intratibial tumors grouped as shown in C (T, tumor; N, the adjacent non‐tumor tissues. Scale bars, 500 µm and 100 µm). ns, not significant; *, *P* < 0.05; **, *P* < 0.01; ***, *P* < 0.001; ****, *P* < 0.0001.

Given that the phosphorylation of ENO1 led to the activation of the MAPK signaling pathway, we further investigated the role of ENO1 phosphorylation in promoting ENZ resistance in PCa cells. For C4‐2B and LNCap cell lines, we stably silenced ENO1 and separately transfected different mutant plasmids. Adding ENZ treatment along with ECM1, we observed that the re‐expression of both shENO1/WT and shENO1/Y189E restored the reduction in cell proliferation caused by ENO1 depletion and rescued ENZ‐induced apoptosis, whereas shENO1/Y189F did not (**Figure** **6**A,B; Figure S5A, Supporting Information). Similar results were observed when PCa cells were treated with docetaxel (Figure S5B,C, Supporting Information).

Immediately after this, we constructed a tumor BM model by generating stable ENO1 knockout C4‐2B cells, followed by transfection with ENO1 plasmids carrying different site‐specific mutations, and subsequently injecting the cells into the tibia of BALB/c‐nu mice. Four weeks later, ENZ was administered to the mice (Figure 6C). In contrast to the shENO1 and shENO1/Y189F groups, where tumor progression slowed, the shENO1/WT and shENO1/Y189E groups exhibited rapid tumor growth and formed larger lesions, which were observed and measured using BLI (Figure 6D) and micro‐CT (Figure 6E; Figure S5D, Supporting Information). Additionally, H&E staining showed that tumor lesions and growth in the shENO1/WT and shENO1/Y189E groups were significantly higher than those in the shENO1 and shENO1/Y189F groups (Figure 6F). Collectively, these data demonstrate that ENO1 phosphorylation is indispensable for ENZ resistance in PCa cells.

### 2.7 PhAH Attenuates ENO1‐Mediated PCa Cell Resistance to ENZ

**Figure 7:**
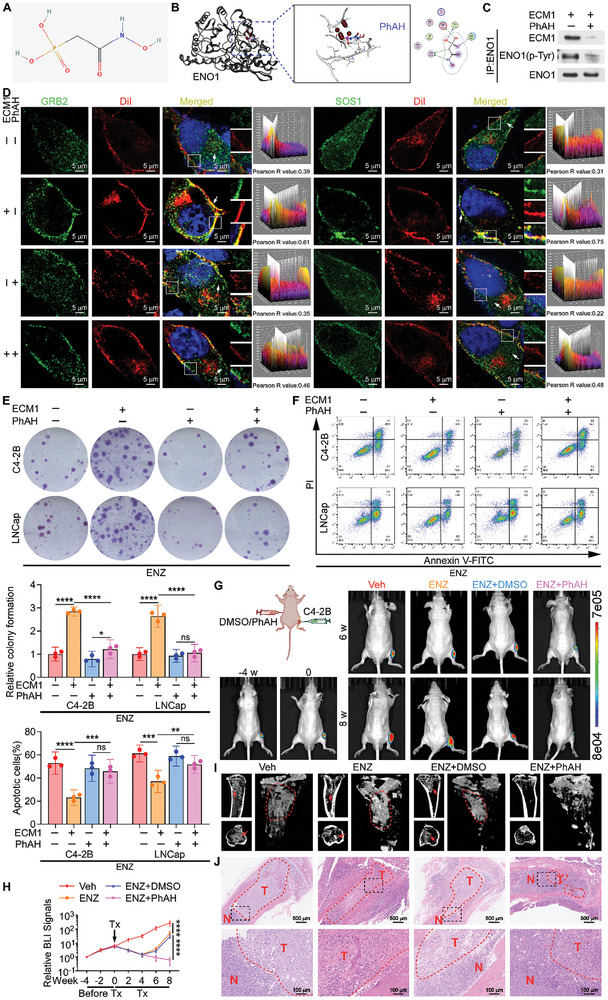
PhAH attenuates ENO1‐mediated PCa cell resistance to ENZ. A) Chemical structure of PhAH. B) Ligand interaction diagram (left) and binding amino acid residue sites (right) of the highest‐scoring PhAH and ENO1 protein molecular docking complex (RMSD = 1.9977; E_score = −3.9538). C) WB detection of the interaction between ECM1 and ENO1, as well as Tyr phosphorylation of ENO1 following immunoprecipitating ENO1 in C4‐2B cells in the presence of ECM1 (200 ng mL^−1^) with or without PhAH (1 µM). D) IF staining and quantification of GRB2, SOS1, and DiI in C4‐2B cells treated with or without ECM1 (200 ng mL^−1^) or PhAH (1 µM). Pearson R value greater than 0.5 indicated co‐localization of the two proteins (Scale bar, 5 µm). E) Representative images (top) and quantification (bottom) of surviving colonies formed by C4‐2B and LNCaP cells treated with or without ECM1 (200 ng mL^−1^) or PhAH (1 µM). F) Flow cytometry analysis showing representative images (top) and quantification (bottom) of apoptosis in C4‐2B and LNCaP cells with or without ECM1 (200 ng mL^−1^) or PhAH (1 µM) treatment. G) Representative BLI of intratibial tumors in mice treated daily oral treatment with Veh, ENZ (20 mg kg^−1^), ENZ (20 mg kg^−1^) combined with intraperitoneal (i.p.) injection of either Veh (DMSO) or PhAH (5 mg kg^−1^) twice weekly at 4 weeks before treatment, and at weeks 0, 6, and 8 during treatment (n = 6 per group). H) Quantification of BLI signals in intratibial tumors of mice before and after Tx as grouped in G (n = 6 per group). I) Representative micro‐CT images of intratibial lesions from mice after 8 weeks of treatment grouped as shown in G (arrows and circles indicate osteoblastic lesions, n = 6 per group). J) Representative H&E images of intratibial tumors grouped as shown in G (T, tumor; N, the adjacent non‐tumor tissues. Scale bars, 500 µm and 100 µm). ns, not significant; *, *P* < 0.05; **, *P* < 0.01; ***, *P* < 0.001; ****, *P* < 0.0001.

Next, we investigated whether a drug or compound could effectively alleviate ENZ resistance by selective inhibition of ENO1 function. The small‐molecule compound phosphonoacetohydroxamate (PhAH, PubChem CID 445375) was obtained from the PubChem Database (https://pubchem.ncbi.nlm.nih.gov), and was identified as a specific inhibitor of ENO1.^[^ ^49^, ^50^, ^51^ ^]^ The chemical structure is shown in **Figure** **7**A. The X‐ray crystal structure of ENO1 (Protein Data Bank [PDB] ID:2PSN) was obtained from the PDB Database (https://rcsb.org). Subsequent docking prediction analysis using molecular operating environment (MOE) 2019.0102 showed that PhAH could effectively bind to the CTD of ENO1with high affinity (Figure 7B; RMSD = 1.9977; E_score = −3.9538), competitively inhibiting ECM1 from binding to ENO1. Furthermore, PhAH inhibited ENO1 phosphorylation induced by ECM1 and activation of the downstream MAPK signaling pathway (Figure 7C; Figure S6A, Supporting Information). IF staining results showed that PhAH prevented ECM1 from recruiting GRB2 and SOS1 to the PCa cell membrane (Figure 7D). Moreover, the promotion of cell proliferation and rescue of apoptosis by ECM1 was reversed by PhAH (Figure 7E,F; Figure S6B, Supporting Information). The inhibitory effect of ECM1 on docetaxel‐induced tumor cell apoptosis was also restored by PhAH (Figure S6C,D, Supporting Information). These results indicate that PhAH blocks the process by which ECM1 promotes tumor cell growth through ENO1.

In addition, in the mouse BM model, mice were treated with ENZ and concurrently received intraperitoneal injections of either dimethyl sulfoxide (DMSO) or PhAH (Figure 7G). Tumor progression was observed through BLI and micro‐CT imaging (Figure 7H,I; Figure S6E, Supporting Information). The results demonstrated that PhAH delayed tumor progression and reduced lesion size (Figure 7J). Taken together, our findings reveal that PhAH, a specific inhibitor targeting ENO1, effectively attenuates ENZ resistance in PCa cells induced by ENO1.

### 2.8 Validation of ECM1/ENO1/MAPK Signaling Axis in Patients with bmCRPC

**Figure 8:**
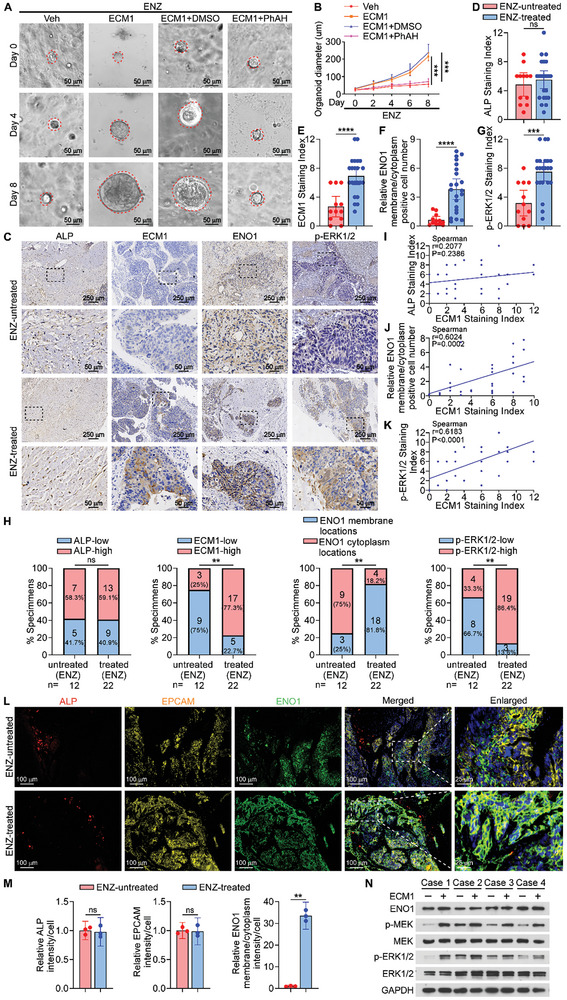
Validation of ECM1/ENO1/MAPK signaling axis in patients with bmCRPC. A) Representative images (left) of PCa PDOs treated with Veh (PBS), ECM1 (200 ng mL^−1^), or ECM1 (200 ng mL^−1^) combined with either DMSO or PhAH (1 µM) in the presence of ENZ (10 µM). B) Assessment of PDOs growth by changes in organoid diameter (right; Scale bar, 50 µm). C) IHC staining of ALP, ECM1, ENO1, and p‐ERK1/2 in ENZ‐untreated (n = 12) or ENZ‐treated (n = 22) bmCRPC patients tissue (Scale bars, 250 µm and 50 µm). D‐G) Staining index quantification of ALP, ECM1, p‐ERK1/2 expression and relative ENO1 membrane/cytoplasm cell number as indicated in C. H) Percentage of specimens showing ENZ‐untreated or treated in relation to the expression levels of ALP, ECM1, p‐ERK1/2 and the membrane or cytoplasm level of ENO1. I‐K) Spearman correlation analysis between the expression levels of ALP, relative ENO1 membrane/cytoplasm cell number, and p‐ERK1/2 with ECM1 expression. L) Representative mIHC staining images of ALP (red, osteoblast marker), EPCAM (yellow, PCa marker), and ENO1 (green) in human bmCRPC tissues (Scale bars, 100 µm and 25 µm). M) Quantification of ALP, EPCAM, and relative ENO1 membrane/cytoplasm intensity per cell in L). N) WB analysis of MEK, p‐MEK, ERK1/2, and p‐ERK1/2 expression in prostate cancer cells extracted from human bmCRPC tissue with or without ECM1 treatment (200 ng mL^−1^). GAPDH was used as the loading control. ns, not significant; *, *P* < 0.05; **, *P* < 0.01; ***, *P* < 0.001; ****, *P* < 0.0001.

We investigated the role of ECM1 in mediating anti‐androgen therapy resistance in a bmCRPC patient‐derived organoids (PDOs) model (**Figure** **8**A). Our results showed that ECM1 promoted ENZ resistance, while the addition of PhAH alleviated this effect of ECM1 (Figure 8B). Furthermore, to evaluate the correlation between the ECM1/ENO1/MAPK signaling axis and resistance in human bmCRPC, we measured the levels of alkaline phosphatase (ALP), ECM1, ENO1, and p‐ERK1/2 in ENZ‐untreated (n = 12) or ENZ‐treated (n = 22) bone metastatic tissues using IHC staining (Figure 8C). Particularly, ALP, a marker of osteoblasts, showed no significant differences in expression between the two groups (Figure 8D). Nevertheless, the expression of ECM1, p‐ERK1/2 and relative ENO1 membrane/cytoplasm cell number were significantly higher in the ENZ‐treated group than in the ENZ‐untreated group (Figure 8E–G). IHC staining results from human bmCRPC specimens revealed that the development of ENZ resistance positively correlated with ECM1, p‐ERK1/2, and membrane ENO1 levels, but not with ALP levels (Figure 8H). Moreover, correlation analysis indicated that ECM1 expression showed no significant correlation with ALP expression (r = 0.2077, *P* = 0.2386; Figure 8I), while positively correlated with relative ENO1 membrane/cytoplasm cell number (r = 0.6024, *P* = 0.0002; Figure 8J) and p‐ERK1/2 expression (r = 0.6183, *P* < 0.0001; Figure 8K). Notably, in ENZ‐treated bmCRPC tissues, multiplex immunohistochemical (mIHC) staining revealed that ENO1 was primarily localized on the PCa cell membrane (Figure 8L,M). In addition, prostate cancer cells extracted from bmCRPC tumor tissue showed activation of the MAPK pathway after treatment with ECM1 (Figure 8N). Overall, our study reveals the molecular mechanism by which osteoblast‐secreted ECM1 in the bone microenvironment under anti‐androgen therapy pressure drives tumor resistance through the ECM1/ENO1/MAPK signaling axis in BMPC.

## 3 Discussion

Research on tumor treatment resistance is extensive and has primarily focused on the mechanisms by which tumor cells develop resistance to therapies. However, tumor cells and their surrounding microenvironment form an integrated whole, and drug treatments can also affect other cells within the microenvironment, potentially mediating the development of tumor resistance.^[^ ^14^ ^]^ The TME significantly influences the fate of tumor cells, with the bone microenvironment being a unique niche that favors colonization, activation, and growth of metastatic tumor cells.^[^ ^21^, ^52^, ^53^ ^]^ Substantial evidence indicates that the immunosuppressive microenvironment formed by immune cells and proteins secreted by CAFs plays a crucial role in the development of tumor resistance.^[^ ^14^, ^22^, ^54^, ^55^ ^]^ However, the impact of bone stromal cells within the microenvironment has received less attention. In our study, osteoblasts from the bone microenvironment secreted ECM1 under ENZ treatment, triggering resistance to anti‐androgen therapy in PCa. Mechanistically, ECM1 interacts with the ENO1 receptor on the prostate cancer cell membrane, leading to its phosphorylation, contributing to the activation of the MAPK signaling pathway, resulting in the development of tumor resistance. Additionally, we found that a specific small‐molecule inhibitor targeting ENO1 significantly weakened ECM1‐induced PCa resistance, potentially offering new avenues for clinical treatment.

BM from PCa is predominantly characterized by osteoblastic lesions with a concurrent osteolytic component, that is co‐regulated by osteoblasts and osteoclasts.^[^ ^56^, ^57^ ^]^ Osteoblasts, a highly specific type of stromal cells, are among the most critical components of the bmCRPC microenvironment. The AR signaling pathway in osteoblasts maintains coordination between bone matrix synthesis, mineralization, and metabolism, thus playing an irreplaceable role in the homeostasis of the bone microenvironment.^[^ ^58^, ^59^ ^]^ However, hormonal therapy for PCa inhibits AR signaling in osteoblasts, and the mechanisms by which osteoblasts influence tumor resistance remain unclear. Notably, Jagged1 derived from osteoblasts activates the Notch signaling pathway, triggering chemotherapy‐resistant BM in breast cancer.^[^ ^17^ ^]^ Thulin et al. reported that during hormonal therapy, osteoblasts mediate the formation of CRPC by regulating the production of steroids in tumor cells, promoting rapid tumor progression.^[^ ^60^ ^]^ Our study showed that under the pressure of the AR pathway inhibitor ENZ, osteoblasts in the microenvironment of BMPC secrete ECM1 protein, which acts on tumor cells, and promotes tumor proliferation and resistance to chemotherapeutic drugs. Our findings reveal the crucial role of osteoblasts, as important components of the bone microenvironment, in inducing treatment resistance in BMPC.

ECM1 is a secreted glycoprotein with various functions, including stimulation of cell proliferation, promotion of angiogenesis, negative regulation of endochondral ossification, and facilitation of tumor progression.^[^ ^61^ ^]^ Our results suggested that the number of osteoblasts surrounding tumor cells in the microenvironment of BMPC showed no significant change before and after the development of ENZ resistance. However, under the influence of the drug, osteoblasts secrete increased levels of ECM1 protein, which binds to the surface of tumor cells and activates the MAPK signaling pathway, promoting cell proliferation and causing treatment resistance. ECM1 has been reported to inhibit mineralization to negatively regulate bone growth and development, consistent with the inhibition of bone maturation by anti‐androgen therapy.^[^ ^62^ ^]^ Moreover, Lee et al. reported that ECM1 could lead to trastuzumab resistance in breast cancer by activating the EGFR/ERK signaling pathway, which is consistent with our findings.^[^ ^18^ ^]^

The ECM1 protein, a non‐cellular structural component of the ECM, promotes tumor invasion and metastasis by interacting with integrin receptors or other cell membrane receptors such as moesin.^[^ ^63^, ^64^ ^]^ Intriguingly, in our study, ECM1 interacted with the ENO1 receptor on the PCa cell membranes. Under normal conditions, ENO1 is a widely distributed protein in the cytoplasm with multiple functions, typically acting as an enolase involved in the glycolysis process. Additionally, ENO1 can function as an RNA‐binding protein (RBP), activate RNA translation, and induce RNA degradation.^[^ ^65^, ^66^ ^]^ Another portion of ENO1 is located on the cell membrane surface, acting as a receptor to bind proteins or small molecules within the ECM to form a multi‐protein complex, thus mediating signal transduction.^[^ ^38^, ^40^, ^67^ ^]^ When ECM1 is coupled to ENO1 on the cell membrane, ENO1 is phosphorylated at Y189. Subsequently, phosphorylated ENO1 is linked to the SH2 domain of GRB2 protein, thereby activating the downstream MAPK signaling pathway and triggering tumor proliferation. Similarly, ENO1 functions as a receptor on the surface of immune cells and binds to apolipoprotein B, aggravating the inflammatory response in rheumatoid arthritis.^[^ ^39^ ^]^ Our study confirmed the non‐metabolic function of ENO1 as a metabolic enzyme, and elucidated its vital role in treatment resistance of PCa.

As most bmCRPC require combination chemotherapy or radiotherapy,^[^ ^25^, ^68^, ^69^ ^]^ it is highly meaningful to identify natural products, drugs, or compounds that can directly or indirectly inhibit ENO1 to block the progression of castration resistance before treatment resistance develops. ENO1 located on the surface of lung cancer cells has been proven to serve as a therapeutic target suitable for clinical applications.^[^ ^70^ ^]^ The study by Chen et al. demonstrates that the ENO1‐specific monoclonal antibody, HuL227, inhibits the growth and invasiveness of prostate cancer cells and reduces inflammatory monocyte recruitment and angiogenesis within the TME.^[^ ^71^ ^]^ Additionally, multiple studies have reported that ENOblock, along with ENO1 autoantibodies, reduces tumor cell growth and migration in various cancers by inhibiting ENO1.^[^ ^38^, ^72^, ^73^ ^]^ Here, we obtained the ENO1 structure from the PDB Database and identified several ENO1 inhibitors in the PubChem Database. Considering that ECM1 connects to the PBD at the C‐terminus of ENO1, we used the MOE software to model and evaluate the 3D binding of the inhibitors with ENO1, and found that PhAH could interact with the C‐terminus of ENO1 with high affinity, competitively antagonizing the binding of ECM1 to ENO1. PhAH is one of the effective and specific inhibitors targeting enolase.^[^ ^49^ ^]^ Our results revealed that PhAH blocked ECM1‐induced promotion of cell proliferation, and enhanced the cytotoxic effect of chemotherapeutic drugs on PCa cells. These findings indicate that targeting ENO1 with PhAH constrains the influence of osteoblasts on PCa cells and increases the sensitivity of tumors to chemotherapy, thereby serving as an effective therapeutic strategy for bmCRPC.

Despite these significant findings, there are some limitations in our study. First, we discovered that osteoblasts produced increased levels of ECM1 protein under ENZ treatment; however, we did not elucidate the specific mechanisms involved. Second, we detected ENO1 phosphorylation at the Y189 site following its interaction with ECM1, but the detailed process remains unclear. Some co‐binding proteins, including tyrosine kinases, may be involved in this process, warranting further investigation. Third, in exploring the cellular components of the bone microenvironment that contribute to ENZ resistance in PCa cells, we did not experimentally verify all cell types, which may have led us to overlook the roles of some cells. Finally, we did not perform high‐throughput screening of all compounds; the binding models predicted by the software did not equate to experimental validation, but could provide valuable insights for further research.

**Figure 9:**
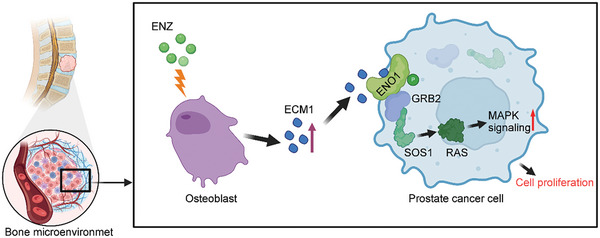
Schematic diagram illustrating that increased osteoblast‐derived ECM1 from the bone microenvironment of BMPC patients induced by ENZ treatment, interacts with the ENO1 receptor on the prostate cancer cell membrane, further recruiting adapter proteins including GRB2 and SOS1, which activates the downstream MAPK signaling pathway to promote the proliferation of PCa cells and induce anti‐androgen resistance.

In summary, our results explain the mechanism of tumor resistance in BMPC following anti‐androgen treatment. Osteoblasts in the BM microenvironment secrete ECM1, which interacts with the ENO1 receptor on the surface of PCa cells and induces ENO1 phosphorylation at the Y189 site, further activating the downstream MAPK signaling pathway, promoting tumor proliferation, and contributing to treatment resistance (**Figure** **9**). This mechanism has not been previously reported. Inhibiting ECM1 or its binding to ENO1 can enhance the efficacy of anti‐androgen therapy. This study highlights the crucial role of ECM1 in driving resistance in bmCRPC. Furthermore, we found that the ENO1 inhibitor PhAH could be a therapeutic option for bmCRPC.

## 4 Experimental Section

### Cell Lines and Culture

The human PCa cell line LNCaP, human osteoblast cell line hFOB1.19, mouse pre‐osteoblastic cell line MC3T3‐E1, mouse pre‐osteoclastic cell line RAW264.7, and mouse fibroblast cell line NCTC clone 929 were obtained from Shanghai Chinese Academy of Sciences Cell Bank (Shanghai, China). C4‐2B and HEK293FT cell lines were purchased from the American Type Culture Collection (ATCC, USA). Mouse bone marrow‐derived mesenchymal stem cell line (mBMSC), and mouse primary osteoblasts (PriOBs) were purchased from Servicebio Technology CO, LTD. (China). Mouse osteocytes, human osteoclasts, human fibroblasts, and human bone marrow‐derived mesenchymal stem cell line (hBMSC) were purchased from Procell Life Science & Technology CO, LTD. (China). C4‐2B and LNCaP were grown in RPMI‐1640 medium (GIBCO; C11875500BT) with the addition of penicillin G (100 U mL^−1^), streptomycin (100 mg mL^−1^) and 10% fetal bovine serum (FBS) (GIBCO; 16250078); NCTC clone 929 and MC3T3‐E1 in Alpha Minimum Essential Medium (αMEM) (Sigma‐Aldrich; M4655); mBMSC, hBMSC, fibroblasts, mouse osteocytes, human osteoclasts, HEK293FT and RAW264.7 in Dulbecco's modified eagle medium (DMEM) (GIBCO; 11965092), and hFOB1.19 in DMEM/F‐12 medium (GIBCO; 11320033). To promote osteoclast formation, RAW264.7 cells were maintained in a complete medium supplemented with 50 ng mL^−1^ recombinant soluble murine M‐CSF (Abcam; ab129146) and 100 ng mL^−1^ recombinant soluble murine RANKL (Abcam; ab129136). Human osteocytes were differentiated from osteoblasts.^[^ ^74^ ^]^ The forementioned cell lines were incubated at 37 °C in a humidified atmosphere with 5% CO2, except for hFOB1.19 at 33.5 °C.

### Plasmids and Transfection

The human complementary DNA (cDNA) targeting ECM1, GRB2, AR, truncated‐ECM1 fragments, and truncated‐GRB2 fragments, were respectively cloned into the pCDH‐CMV‐MCS‐puro plasmid vector (System Biosciences; CD500B‐1) tagged with the Flag peptide sequence for overexpression, ENO1, truncated‐ENO1 fragments, ENO1‐WT, ENO1‐Y189F, and ENO1‐Y189E were respectively cloned into the pCDH‐CMV‐MCS‐puro plasmid vector (System Biosciences; CD500B‐1) tagged with the HA peptide sequence for overexpression. Short hairpin RNA (shRNAs) against ECM1, ENO1, GRB2, and SOS1 were individually cloned into the pLKO.1‐puro lentiviral vector (Addgene; 8453). shRNA or plasmids transfection was performed using Lipofectamine 3000 reagent (Thermo Fisher Scientific; L3000015) following the manufacturer's guidelines. Stable PCa cell lines expressing AR, ECM1, truncated‐ECM1 fragments, GRB2, truncated‐GRB2 fragments, GRB2^WT^, GRB2^mut^, ENO1, truncated‐ENO1 fragments, ENO1‐WT, ENO1‐Y189F and ENO1‐Y189E, as well as those expressing sh‐ECM1, sh‐ENO1, sh‐GRB2, or sh‐SOS1, were generated by infecting HEK293FT cells with filtered lentivirus containing the respective constructs. The cells were subsequently selected using 0.5µg mL^−1^ puromycin (Sigma Aldrich; P8833) for a duration of 10 days. The transfection efficiency was confirmed through western blotting. The shRNA targeting sequences were as follows: sh‐ECM1#1 (5’‐ CAACTGCTTCAACATCAATTA‐3′). sh‐ECM1#2 (5′‐ CGCTGCTGTGACCTGCCATTT‐3′). sh‐ENO1#1 (5′‐CGTGAACGAGAAGTCCTGCAA‐3′). sh‐ENO1#2 (5′‐ CCACTGTTGAGGTTGATCTCT‐3′). sh‐GRB2#1 (5′‐ GCAGAAGAAATGCTTAGCAAA‐3′). sh‐GRB2#2 (5′‐ GATCTACATCTGTCTCCAGAA‐3′). sh‐SOS1#1 (5′‐ GCACTTTATTTGCAGTCAATA‐3′). sh‐SOS1#2 (5′‐ CCACAGATGTTTGCAGTGTAT‐3′) (synthesized by Sigma).

### RNA Extraction, Reverse Transcription, and Real‐Time Quantitative PCR (qRT‐PCR)

As reported previously,^[^ ^59^ ^]^ TRIzol reagent (Invitrogen; 15596018) was utilized to extract total RNA. To synthesize cDNA, 2 µg of RNA from each sample was reverse‐transcribed using Color Reverse Transcription Kit (EZBioscience; A0010CGQ) according to the manufacturer's instructions. The cDNA was amplified and quantified using 2× Color SYBR Green qPCR Master Mix (EZBioscience; A0012‐R2) on a CFX96 system (Bio‐Rad, USA). For normalization, glyceraldehyde‐3‐phosphate dehydrogenase (GAPDH) served as an internal control. The primer sequences are provided in Table S1 (Supporting Information). Relative gene expression changes were calculated using the comparative 2‐ΔΔCt method.^[^ ^75^ ^]^

### Western Blotting (WB)

Membrane and cytoplasmic fractions were separated using the Membrane and Cytosol Protein Extraction Kit (Beyotime; P0033) following the manufacturer's instructions, and whole‐cell lysates were obtained using radioimmunoprecipitation assay (RIPA) buffer (Cell Signaling Technology; 9806S). A Pierce BCA Protein Assay kit (Thermo Fisher Scientific; 23227) was used to quantify protein concentrations. Western blotting was performed as previously detailed.^[^ ^76^ ^]^ The primary antibodies utilized for protein detection included ENO1 Rabbit pAb (Proteintech; 11204‐1‐AP, 1:2000), ECM1 Rabbit pAb (Proteintech; 11521‐1‐AP, 1:1000), GRB2 Rabbit mAb (Abcam; ab32037, 1:5000), SOS1 Rabbit pAb (Proteintech; 55041‐1‐AP, 1:1000), HA‐Tag Rabbit mAb (Cell Signaling Technology; #3724, 1:1000), His‐Tag Rabbit mAb (Cell Signaling Technology; #2365, 1:1000), Flag‐Tag Rabbit mAb (Cell Signaling Technology; #14793, 1:1000), MEK Rabbit mAb (Cell Signaling Technology; #8727, 1:1000), Phospho‐MEK (Ser217/Ser221) Rabbit mAb (Cell Signaling Technology; #9154, 1:1000), ERK1/2 Rabbit mAb (Cell Signaling Technology; #4695, 1:1000), Phospho‐ERK1/2 (Thr202/Tyr204) Rabbit mAb (Cell Signaling Technology; #4370, 1:2000), EGFR Rabbit mAb (Cell Signaling Technology; #4267, 1:1000), Phospho‐EGFR (Tyr978) Rabbit mAb (Cell Signaling Technology; #3790, 1:1000), IGF1R Rabbit mAb (Cell Signaling Technology; #9750, 1:1000), Phospho‐IGF1R Rabbit mAb (Cell Signaling Technology; #3918, 1:1000), FGFR1 Mouse mAb (Proteintech; 60325‐1‐Ig, 1:1000), Phospho‐FGFR1 (Tyr653/Tyr654) Rabbit pAb (Sigma Aldrich; 06–1433, 1:1000) and Phospho‐Tyrosine Mouse mAb (P‐Tyr‐100) (Cell Signaling Technology; #9411, 1:2000). GAPDH Rabbit mAb (Cell Signaling Technology; #2118, 1:1000) and plasma membrane Ca2+ ATPase 1 (PMCA1) Rabbit mAb (Abcam; ab190355, 1:1000) were used as internal controls.

### Hematoxylin‐Eosin (H&E), Immunohistochemistry (IHC) Staining, and Scoring

The tibiae of nude mice were fixed with a 4% paraformaldehyde solution overnight. Subsequently, the bones underwent decalcification in a 5% EDTA solution for a week before being embedded in paraffin wax. Sections were either stained with hematoxylin and eosin. IHC staining of human bone metastatic PCa tissues and mouse tumors was conducted as described in our previous study.^[^ ^77^ ^]^ Paraffin‐embedded (FFPE) specimens were cut into 4‐µm slices and baked at 65 °C for 30 min. The sections were deparaffinized with xylene and rehydrated. Antigen retrieval was performed by immersing the sections into an EDTA antigenic retrieval buffer and subjecting them to microwave treatment. The samples were subsequently treated with 3% hydrogen peroxide in methanol to inhibit the endogenous peroxidase activity. Then, non‐specific binding was blocked by incubating the sections with 1% bovine serum albumin, and then incubated overnight with primary antibodies at 4 °C. After thorough washing, the tissue sections were exposed to a biotinylated anti‐rabbit secondary antibody, followed by incubation with a streptavidin‐horseradish peroxidase complex (ZsBio, China). Finally, the sections were stained with 3‐amino‐9‐ethyl carbazole, counterstained with 10% Mayer's hematoxylin, dehydrated, and mounted on a Crystal Mount. The primary antibodies utilized for the incubation of the tissue sections included: ALP Rabbit pAb (Affinity; #DF6225, 1:100), ECM1 Rabbit pAb (Proteintech; 11521‐1‐AP, 1:200), ENO1 Rabbit pAb (Proteintech; 11204‐1‐AP, 1:500), Phospho‐ERK1/2 (Thr202/Tyr204) Rabbit mAb (Cell Signaling Technology; #4370, 1:200).

The staining index (SI) was calculated as the staining intensity score multiplied by the proportion of positive tumor cells score. Two independent pathologists blinded to the clinical outcomes evaluated the SI. The staining intensity was graded on a scale ranging from 0 (no staining) to 3 (brown), with intermediate values of 1 (light yellow) and 2 (yellow‐brown). The proportion of positive tumor cells was scored using the following criteria: 0 (no positive tumor cells), 1 (<10% positive tumor cells), 2 (10%–35% positive tumor cells), 3 (36%–70% positive tumor cells), and 4 (>70% positive tumor cells). Samples with an SI of ≥ 6 were classified as having high expression, whereas those with an SI of < 6 were considered to have low expression. Specifically, for the ENO1 marker, the ratio of membrane‐positive to cytoplasm‐positive cells in 5 randomly selected fields from at least 3 independent experiments was used as the evaluation metric and analyzed by ImageJ software (version 2.0.0, NIH, USA).

### Immunofluorescence (IF) Staining

For IF staining, the indicated cells were seeded onto coverslips in 24‐well plates and cultured accordingly. The coverslips were gently washed thrice with PBS, fixed with 4% paraformaldehyde for 15 min, permeabilized with 1% Triton X‐100 for 20 min, and blocked with 5% Bovine Serum Albumin (BSA) for 30 min at room temperature. Subsequently, cells were respectively incubated overnight at 4 °C with primary antibodies including ECM1 Rabbit pAb (Proteintech; 11521‐1‐AP, 1:250), ENO1 Mouse mAb (Thermo Fisher Scientific; MA5‐47393, 1:2000), GRB2 Rabbit pAb (Proteintech; 10254‐2‐AP, 1:50) or SOS1 Rabbit pAb (Proteintech; 55041‐1‐AP, 1:100). The next day, Alexa Fluor 488 conjugate goat anti‐mouse IgG (H+L) (Cell Signaling Technology; #4408, 1:500) or Alexa Fluor 555 conjugate goat anti‐rabbit IgG (H+L) (Cell Signaling Technology; #4413, 1;500) was used as the secondary antibody and incubated with cells at room temperature for 1 h. Specifically, the cell membrane was stained with the red fluorescent probe DiI (Beyotime; C1036). Then nuclei were counterstained using DAPI (Sigma‐Aldrich; 28718‐90‐3) for 10 min. All the images were acquired using a Nikon Eclipse Ti fluorescence microscope equipped with a Nikon DS‐Qi2 monochrome camera (version 5.21.00; Nikon, Tokyo, Japan). The mean fluorescence intensity was quantitatively analyzed using ImageJ software (version 2.0.0, NIH, USA). Pearson R value greater than 0.5 indicated co‐localization of the two proteins.

### Multiplex Immunohistochemical (mIHC) Staining

Tumor tissue slides were subjected to incubation with ALP Rabbit pAb (Affinity; #DF6225, 1:100), EPCAM Rabbit mAb (Abcam; ab223582), ENO1 Rabbit mAb (Abcam; ab227978) and then were stained with the PANO Reagents PPD650, PPD570, and PPD520 using a Multi‐fluorescence Immunohistochemistry Kit 4 Color TSA‐Rab‐275 (Panovue; #10079100020) according to the manufacturer's instructions. Nuclei were counterstained using DAPI (Sigma‐Aldrich; 28718‐90‐3). The fluorescent signals were detected using a Zeiss LSM710 confocal microscope (Carl Zeiss, Oberkochen, Germany). The average fluorescence intensity was measured across over 5 randomly selected fields or 50 cells in at least 3 independent experiments, and further analyzed by ImageJ software (version 2.0.0, NIH, USA). Specifically, for the ENO1 marker, the membrane‐to‐cytoplasm signal intensity ratio within each cell was used as the evaluation metric.

### Co‐Immunoprecipitation (co‐IP) Assay

HEK293FT cells were co‐transfected with the respective plasmid DNAs and incubated for 48 h. Immunoprecipitation assays were conducted using HEK293FT, C4‐2B, and LNCaP cells. After washing twice with pre‐cooled PBS, the indicated cells were lysed in cold lysis buffer containing phenylmethanesulfonyl fluoride (PMSF) (Beyotime; ST507, 1:100) and a protease/phosphatase inhibitor cocktail (Cell Signaling Technology; #5872, 1:100). The lysates were then incubated with target antibodies and protein A/G‐conjugated magnetic beads (Thermo Fisher Scientific; #88802) overnight at 4 °C. The antibodies used for the IP of target proteins included Flag‐Tag Rabbit mAb (Cell Signaling Technology; #14793, 1:50), ECM1 Mouse mAb (Thermo Fisher Scientific; MA1‐19051, 1:50) and ENO1 Mouse mAb (Thermo Fisher Scientific; MA5‐49512, 1:100). Magnetic beads containing affinity‐bound proteins were rinsed six times with RIPA buffer. The eluates were analyzed by western blotting. Alternatively, cell lysates separately underwent direct incubation with anti‐Flag or anti‐HA magnetic beads (Selleck; B26101 or B26201) for 4 h at 4 °C. The beads were then separated using a magnet. After heat denaturation, both the input and co‐IP samples were subjected to WB analysis using anti‐HA or anti‐Flag antibodies as described above. To minimize background noise, the interacting proteins were analyzed using primary antibodies derived from different biological hosts and compared with those used for IP.

To detect ENO1 phosphorylation, PCa cell lysates were used for IP with ENO1 Rabbit pAb (Sigma‐Aldrich; SAB5700028, 1:100). Lysates from 293FT cells transfected with various HA‐ENO1 constructs were subjected to IP using HA‐affinity magnetic beads (Selleck; B26201). Subsequently, the eluates obtained from these immunoprecipitates were analyzed by western blotting using a Phospho‐Tyrosine Mouse mAb (P‐Tyr‐100) (Cell Signaling Technology; #9411, 1:2000).

### Proximity Ligation Assay (PLA)

The proximity ligation assay was carried out using a Rabbit PLUS and Mouse MINUS Duolink in situ PLA kit (Sigma‐Aldrich; DUO92102) in accordance with the manufacturer's instructions. In brief, the indicated cells on coverslips were fixed with 4% formaldehyde for 15 min at room temperature; then washed with TBS and blocked for 1 h with Duolink Blocking Solution in a humidified chamber. Subsequently, the coverslips were incubated overnight at 4 °C with anti‐Flag (Sigma‐Aldrich, F1804, mouse) and anti‐ENO1 (Abcam; ab227978, rabbit) antibodies. After thorough washing with TBST, the proximity ligation reaction was performed using the PLA kit (Sigma‐Aldrich; DUO92102). Nuclei were counterstained with DAPI (Sigma‐Aldrich; 28718‐90‐3) for visualization. PLA signals were detected using an Olympus BX51 microscope (Olympus, Tokyo, Japan) with a 40x objective, and further analyzed using ImageJ software (version 2.0.0, NIH, USA). The PLA signal was quantified by counting the number of foci per cell across 5 randomly selected fields.

### Colony Formation Assay

A colony formation assay was performed to assess cell viability. The cells were seeded at a density of 10^3^ cells per well in 6‐well plates. Following attachment, the cells were subjected to different treatments according to their respective assigned groups. Two weeks later, cells were fixed with 4% paraformaldehyde and stained with 0.2% crystal violet. Clonogenic ability was evaluated by quantifying the number of colonies formed. Images of the plates were analyzed using ImageJ software (version 2.0.0, NIH, USA).

### Cell Counting Kit‐8 (CCK‐8) Assay

Cells were plated in 96‐well plates and treated with different reagents. Each well was added with 10 µL solution (Beyotime; C0038) and then incubated in darkness at 37 °C for 2 h. Cell viability was expressed as the percentage of surviving cells in drug‐treated cells relative to vehicle‐treated cells and evaluated daily for a total of 7 days. An absorbance (450 nm) was measured using Microplate Spectrophotometer (Bio‐Tek, USA) in accordance with the manufacturer's protocol.

### 5‐Ethynyl‐2’‐Deoxyuridine (EdU) Assay

The EdU assay was performed to assess cellular proliferation using the Cell‐Light EdU Apollo567 In Vitro Kit (RiboBio; C10310‐1), following the manufacturer's protocol. After treatment with reagents for 48 h, cells were incubated with 10 µM EdU for 2 h before being fixed with 4% paraformaldehyde. Following permeabilization with 0.3% Triton X‐100, the cells were stained with the Apollo reaction solution. The cell nuclei were stained with Hoechst 33342 for 30 min. All images were acquired using a research‐grade inverted fluorescence microscope (OLYMPUS IX73, Tokyo, Japan). EdU‐positive cells were quantified as follows: (number of EdU‐labeled cells/number of Hoechst 33342‐stained cells) × 100. Images were analyzed using ImageJ software (version 2.0.0, NIH, Bethesda, MD, USA).

### Chemical Reagents

ENZ (MCE; HY‐70002) contained 200 mg of lyophilized enzalutamide powder per bottle. ENZ dissolved in DMSO (Sigma‐Aldrich; D4540) was used at a concentration of 10 µM for treating human PCa cell lines and administered orally to BALB/c‐nu mice with a dosage of 20 mg kg^−1^.^[^ ^78^ ^]^ Each bottle of docetaxel (MCE; HY‐B0011) contained 200 mg of lyophilized docetaxel powder. After dissolution in DMSO, the final concentration used to treat human PCa cell lines was 10 µM.^[^ ^79^, ^80^ ^]^ Recombinant human protein ECM1 (R&D; 3937‐EC‐050), LGALS3BP (Abcam; ab132454), and HMGB1 (Abcam; ab20339) were used for subsequent experiments. The working concentration of ECM1, LGALS3BP, and HMGB1 proteins was 200 ng mL^−1^.^[^ ^81^ ^]^ The proteinase K (MCE; HY‐108717) was utilized at a concentration of 200 µg mL^−1^.^[^ ^13^ ^]^ ECM1 monoclonal antibody (Thermo Fisher Scientific; 1893‐MSM2‐P1; 5µg mL^−1^) was used to interact with ECM1 protein. Mouse ECM1 affinity‐purified polyclonal antibody (pAb) (R&D; AF4428) was administered to BALB/c‐nu mice via tail vein injection at a concentration of 50 mg kg^−1^. The working concentration of Dihydrotestosterone (APExBIO; #B8214‐25; DHT) is 1nM. The Ras inhibitor MCP110 (MCE; HY‐123673), MEK inhibitor U0126 (MCE; HY‐12031A), ERK inhibitor ulixertinib (MCE; HY‐15816), EGFR inhibitor lapatinib (MCE; HY‐50898), FGFR1 inhibitor fexagratinib (MCE; HY‐13330), and IGF1R inhibitor linsitinib (MCE; HY‐10191) required dissolution in DMSO before use. Phosphonoacetohydroxamate (PhAH, PubChem CID 445375), an ENO1 targeted inhibitor (MCE; HY‐W647074), was purchased for use as a competitive binder at the ENO1 site.

### Quantitative Co‐Culture Assays and Transwell System Model

GFP‐tagged C4‐2B cells were co‐cultured individually with osteocytes, osteoblasts, osteoclasts, mesenchymal stem cells, or fibroblasts, and seeded in black‐walled 96‐well plates containing type I collagen. ENZ (10µM) was added with fresh culture medium and drugs were replaced every three days. Images were captured every two days using a ZEISS Vert. A1 microscope (Carl Zeiss, Oberkochen, Germany), and fluorescence intensity were quantified using Image‐Pro Plus (version 6.0; Media Cybernetics Inc., USA). At least two duplicate wells were used for the assessment.

A Transwell system model was established to prevent direct cell‐cell contact. PCa cells were seeded at varying densities in the lower chambers of a 6‐well plate based on the specific requirements of different assays. A culture insert system with a 0.4‐µm pore size permeable membrane (Corning; 3412) was then placed into each well. Subsequently, 5 × 10^5^ osteoblasts were cultured in the upper chamber of the Transwell. The culture system was maintained in a growth medium appropriate for both cell types. Cell viability of PCa cells was assessed using the CCK‐8 assay.

### Conditioned Medium Collection

A total of 5×10^6^ osteoblasts were cultured in a 10 cm culture dish. After cell adhesion, ENZ treatment was applied for 72 h. The cells were then rinsed three times with PBS and the medium was replaced with a serum‐free medium. The first batch of CM was collected 48 h later and fresh serum‐free medium was added to the cells. The collected CM was filtered through a 0.45 µm filter (Millipore; SLHA033SS) to remove cell debris, and stored at 4 °C. After 48 h, a second batch of CM was collected and filtered similarly. Both batches of CM were combined and concentrated in Vivaspin protein ultrafiltration centrifuge tubes (Sartorius; VS15T02) for subsequent detection or purification.

### Flow Cytometry Analysis

Apoptosis was detected by flow cytometry using the Annexin V‐FITC/PI Apoptosis Detection Kit (Vazyme; A211‐02) according to the manufacturer's instructions. Cells were grown in ultra‐low attachment plates for 48 h, then harvested and centrifuged at 1200 × g for 5 min at 4 °C. After washing twice with pre‐cooled PBS, cells were resuspended with 100µL 1 × binding buffer and subsequently incubated with 5µL Annexin V‐FITC and 5µL Propidium iodide (PI) Staining Solution for 10 min at room temperature. Then 400 µL 1 × binding buffer was added to samples. Immediately following this, samples were analyzed on a CytoFLEX Flow Cytometer (Beckman, USA), and the data were analyzed using FlowJo software (V10.6.2, BD, USA).

### Enzyme‐Linked Immunosorbent Assay (ELISA)

Cells were cultured in 6‐well plates, and then subjected to appropriate treatments for 24 h in a standard cell culture incubator at 37 °C with 5% CO2. The cells were washed twice with pre‐chilled PBS on ice and centrifuged at 500 g for 5 min to collect the supernatant. The cell supernatants were analyzed for ECM1 using a Mouse ECM1 ELISA kit (RayBio; ELM‐ECM1‐1) according to the manufacturer's protocol. The data were read on a TECAN Multifunctional Enzyme Labeler (TECAN, Switzerland) at a wavelength of 450 nm. The ECM1 concentration was determined by extrapolating standard curve based on the absorbance of the samples.

### Luciferase Reporter Assay

Cells were plated in 24‐well plates and reached a proliferation rate of 60–80% confluence after 24 h. The pGL3‐ECM1‐promoter and Renilla luciferase vector were co‐transfected into MC3T3‐E1 and hFOB1.19 cells. After treatment with vehicle or DHT for 48 h, luciferase activities were detected using a Dual‐Luciferase Reporter Assay kit (Promega; E1910) according to the manufacturer's instructions. The luciferase activity of each cell lysate was standardized based on the Renilla luciferase activity.

### Chromatin Immunoprecipitation (ChIP) Assays

ChIP assays were implemented using ChIP Kit (Abcam; ab500). In brief, cells were treated with vehicle or DHT for 48 h, and fixed with 1% formaldehyde to cross‐link proteins to DNA. After ceasing the cross‐linking reaction with glycine for 5 min, DNA was broken into small fragments by sonication. 10 µL of the sample was collected for use as the input group. The remaining supernatant was incubated overnight at 4 °C with AR Rabbit mAb (Cell Signaling Technology; #5153, 1:100), or anti‐IgG antibody (Cell Signaling Technology; #2729, 1:50) using ChIP‐Grade Protein G Magnetic Beads (Cell Signaling Technology; #9006). Then, the cross‐linked DNA‐Protein complexes were eluted using ChIP Elution Buffer and the crosslinks were reversed by incubating at 65 °C for 1 h. Subsequently, DNA was purified using a centrifugal column and analyzed by PCR. The primer sequences for ECM1 were listed below: Forward (5′‐ GAGCAGAAACTCCTCAGCCA‐3′), Reverse (5′‐ AGGGCTGGATTCACAGAGTG‐3′).

### Liquid Chromatography/Tandem Mass Spectrometry (LC‐MS/MS) Analysis

Osteoblasts, including MC3T3‐E1 and hFOB1.19 cells were treated with either vehicle or ENZ for 72 h. Subsequently, cell culture supernatants were collected and subjected to LC‐MS/MS analysis to identify differential proteins.

Recombinant human ECM1 tagged with Flag was added to C4‐2B cells and incubated for 48 h. After washing twice with pre‐chilled PBS, indicated cells were lysed to isolate membrane proteins, and then separately incubated overnight at 4 °C with anti‐IgG magnetic beads (Beyotime; P2173) or anti‐Flag magnetic beads (Beyotime; P2115). After washing six times with RIPA buffer, the protein samples were subjected to MS analysis to identify the binding proteins of ECM1.

For MS analysis of endogenous ENO1 phospho‐peptides, C4‐2B cells were treated with vehicle or recombinant human ECM1 protein for 48h. ENO1 protein was immunoprecipitated from C4‐2B cell lysates using protein A/G‐conjugated magnetic beads (Thermo Fisher Scientific; #88802) and ENO1 Mouse mAb (Thermo Fisher Scientific; MA5‐49512, 1:100). Subsequently, samples were washed six times with RIPA buffer supplemented with a protease/phosphatase inhibitor cocktail (Cell Signaling Technology; #5872, 1:100). MS analysis was conducted to identify the phosphorylated peptide segments of the ENO1 protein and the corresponding phosphorylation sites.

The MS analysis and subsequent interpretation were performed by Shenzhen Wininnovate Biotechnology Co., Ltd. (China).

### RNA Sequencing (RNA‐Seq)

C4‐2B cells were cultured in 6‐well plates and treated with vehicle (n = 3), ENZ (n = 3), ECM1 (n = 3), or ENZ+ECM1 (n = 3) for 48 h. Subsequently, total RNA was extracted and subjected to RNA sequencing (RNA‐seq). Genes exhibiting differential expression between vehicle and ECM1, as well as between ENZ and ENZ+ECM1, were identified. Kyoto Encyclopedia of Genes and Genomes (KEGG) pathway enrichment analysis and Gene Set Enrichment Analysis (GSEA) were conducted to elucidate functional pathways.

### Animal Study

Ethical approval for animal experiments was provided by the Institutional Animal Care and Use Committee of Sun Yat‐sen University. The ethics approval number for the animal experiments is NO.SYSU‐IACUC‐2023‐001209. C4‐2B cells were suspended in 100 µL of PBS at a concentration of 2 × 10^7^ cells per 100 µL. After anesthetizing male BALB/c‐nu mice aged 4–6 weeks with isoflurane, 10 µL of the cell suspension was slowly injected into the tibia using a 29‐G insulin syringe (BD; 320310) with a drilling motion to construct the intratibial injection model. For the subcutaneous tumor model, 100 µL of cell suspension containing 2 × 10^6^ cells was injected subcutaneously into the mice. Mice were monitored every three days for bone metastases using BLI. Subcutaneous tumor volumes were measured weekly, and determined using the following formula: length (L)×width (W)×width (W)/2.^[^ ^28^ ^]^ For the drug treatment experiments, enzalutamide (20 mg kg^−1^) or vehicle was administered daily via oral gavage. Mouse ECM1 affinity‐purified pAb (R&D; AF4428) was administered at a dose of 50 mg kg^−1^ twice weekly through tail vein injection. Recombinant human protein ECM1 (R&D; 3937‐EC‐050) was injected into mouse subcutaneous tumors (1 µg/2 × 10^6^ cells). PhAH (5 mg kg^−1^) or vehicle was also given via intraperitoneal injection twice weekly. The drug administration process started four weeks after tumor implantation and lasted for eight consecutive weeks. The mice were euthanized based on their survival time or upon reaching the observation endpoint.

### Micro‐CT Analysis

The hind limbs of euthanized mice were removed and immersed in a 4% paraformaldehyde solution. The fixed hind limbs were scanned using a micro‐CT scanner (Bruker SkyScan 1276, Belgium). NRecon software was used to reconstruct the 3D models. A Dataviewer was utilized to observe the coronal, sagittal, and transaxial sections. Following the digital removal of the cortical bone, the trabecular volume of interest was identified, starting from the metaphysis and encompassing all trabeculae within a 1 mm^3^ region. Bone‐related parameters were measured and analyzed using CTAn and CTVol.

### Patient‐Derived Organoids (PDOs) Experiments

The use of patient samples was approved by the Clinical Research and Experimental Animal Ethics Committee of the First Affiliated Hospital of Sun Yat‐sen University (No. [2023]617). This study was conducted in accordance with the Declaration of Helsinki, and all participating patients provided informed consent. Samples were acquired during the necessary procedures, and the patients received no compensation for this study.

The bmCRPC tumor samples obtained from surgical resection were preserved in advanced DMEM/F12 serum free medium (Thermo Fisher Scientific; 12634010) supplemented with 10 mM Hepes (Thermo Fisher Scientific; 15630080), 10 µM Y‐27632 (Selleck, S6390), and 100 µg mL^−1^ Primocin (Thermo Fisher Scientific; ant‐pm). After being washed three times with Hank's balanced salt solution (GIBCO; 88284), the samples were minced into 1 mm^3^ pieces, followed by incubation in a digestion solution composed of the basic medium, collagenase type I (Gibco; 17100‐017, 1:100), collagenase type II (Gibco; 17101‐015, 1:100), and TrypLE Express Enzyme (Gibco; 12605010) at 37 °C for 30 min, with intermittent gentle mixing every 6 min. Digestion was terminated by adding a pre‐chilled advanced DMEM/F12 serum free medium. The suspension was then filtered through a 100 µm cell strainer into a 50 mL centrifuge tube, followed by centrifugation at 1200g for 5 min. After discarding the supernatant, the cell pellets were mixed with pre‐chilled Cultrex Basement Membrane Extract (BME) (R&D; 3432‐001‐01) on ice, with a volume ratio of BME of no less than 70%. The BME‐cell suspension was quickly dispensed into pre‐warmed 48‐well plates. The plates were subsequently placed in an upright position in a 37 °C incubator for 5 min, followed by inversion for 30 min. Once the cell mixture solidified, Prostate Cancer Organoid Medium (Accuroid; M117) was added to each well. The culture medium was refreshed every two days, and the organoids were sub‐cultured weekly using a combination of mechanical force and enzymatic digestion. After drug treatment, the organoids were photographed and various parameters were measured daily using a Nikon Eclipse Ti fluorescence microscope equipped with a Nikon DS‐Qi2 monochrome camera (version 5.21.00; Nikon, Tokyo, Japan).

As reported in our previous study, prostate cancer cells were extracted from bone metastases in bmCRPC patients.^[^ ^82^ ^]^ The extracted prostate cancer cells were treated with ECM1 for 48 h, followed by WB analysis to detect the activation of the MAPK signaling pathway.

### Molecular Docking Analysis and Visualization

The AlphaFold Protein Structure Database (https://alphafold.com) was used to predict the structures of ECM1 and GRB2 proteins. The quality of the models was evaluated using the predicted aligned errors. The X‐ray crystal structure of ENO1 (Protein Data Bank [PDB] ID:2PSN) was retrieved from the PDB Database (https://rcsb.org). Molecular dynamics docking simulations between ECM1 and ENO1, as well as ENO1 and GRB2, were conducted using ClusPro 2.0 (https://cluspro.org).^[^ ^43^ ^]^ Interaction mode analysis and visualization of the docking results were performed using PyMOL 2.5.0. The 3D structure of PhAH in SDF format was obtained from the PubChem Database (https://pubchem.ncbi.nlm.nih.gov). The structures of ENO1 and PhAH were imported into the Molecular Operating Environment (MOE) 2019.0102 for predicting, analyzing, and visualizing the molecular docking models.

### Statistical Analysis

Statistical analysis was performed with GraphPad Prism 8.0.2 (GraphPad Inc., USA). Data are presented as mean ± standard deviation (SD). Quantitative experiments were performed at least three times in an independent manner. Quantitative data were analyzed using Student t‐test for normally distributed data with two groups, Mann–Whitney U test for non‐normally distributed data with two groups and one‐way or two‐way analysis of variance (ANOVA) for multiple groups. Categorical variables and constituent ratios were evaluated using the χ2 test. The correlation between two parameters was identified by Spearman rank correlation analysis. Statistical significance was set at *P* < 0.05 (two‐tailed). All statistically significant values shown in the Figures are denoted as follows: ns, not significant; *, *P* < 0.05; **, *P* < 0.01; ***, *P* < 0.001; ****, *P* < 0.0001.

### Ethics Approval and Consent to Participate

Ethical approval for animal experiments was provided by the Institutional Animal Care and Use Committee of Sun Yat‐sen University. The ethics approval number for the animal experiments is NO.SYSU‐IACUC‐2023‐001209. The use of patient samples was approved by the Clinical Research and Experimental Animal Ethics Committee of the First Affiliated Hospital of Sun Yat‐sen University (No. [2023]617). All participating patients provided informed consent.

## Conflict of Interest

The authors declare no conflict of interest.

## Author Contributions

X.W., M.W., Q.L., and L.H. contributed equally to this work. Y.D., X.P., C.L., and X.W. developed the original idea and designed the experiments. X.W. prepared the Figures and drafted the manuscript. X.W., M.W., Q.L., L.H., B.Z, X.C., and G.C. conducted the experiments and contributed to data analysis. Y.D., H.D., and X.P. provided critical reagents and/or clinical samples. Y.D. and X.P. supervised the study. All authors contributed to revising the manuscript and approved the final version for publication.

## Supporting information



Supporting Information

## Data Availability

The data that support the findings of this study are available from the corresponding author upon reasonable request.
