# Supplementary material for: Osteoblast‐Derived ECM1 Promotes Anti‐Androgen Resistance in Bone Metastatic Prostate Cancer
Source: Adv Sci (Weinh). 2024 Nov 20;12(2):2407662. doi: 10.1002/advs.202407662 (PMC11727142; doi:10.1002/advs.202407662)
Supplement: Supplementary file 1 — Supporting Information [file ADVS-12-2407662-s001.docx]

Supporting Information for

**Osteoblast-derived ECM1 Promotes Anti-androgen Resistance in Bone Metastatic Prostate Cancer**

*Xinwen Wang,*^1,2#^ *Min Wang,*^2,3#^ *Qijun Lin,*^1,2#^ *Lixin He,*^4#^ *Baolin Zhang,*^1,2^ *Xin Chen,*^1,2^ *Guanhong Chen,*^1,2^ *Hong Du,*^3^ *Chuandong Lang,*^5*^ *Xinsheng Peng,*^1,2*^ *and Yuhu Dai,*^1,2*^

^1^Department of Orthopedic Surgery, the First Affiliated Hospital, Sun Yat-Sen University, Guangzhou 510080, China

^2^Guangdong Provincial Key Laboratory of Orthopedics and Traumatology, Guangzhou 510080, China

^3^Department of Pathology, Guangzhou First People’s Hospital, Guangzhou 510080, China

^4^Department of Experimental Research, State Key Laboratory of Oncology in South China, Collaborative Innovation Center for Cancer Medicine, Sun Yat-sen University Cancer Center, Guangzhou 510060, China

^5^Department of Orthopedics, The First Affiliated Hospital of USTC, Division of Life Sciences and Medicine, University of Science and Technology of China, Hefei 230001, China

^#^**These authors contributed equally to this work.**

^*^**Correspondence to:**

Yuhu Dai, and Xinsheng Peng, Department of Orthopedic Surgery, the First Affiliated Hospital of Sun Yat-Sen University, 58# Zhongshan 2rd Road, 510080 Guangzhou, Guangdong Province, China.

Chuandong Lang, Department of Orthopedics, The First Affiliated Hospital of USTC, Division of Life Sciences and Medicine, University of Science and Technology of China, 17# Lujiang Road, 230001 Hefei, Anhui Province, China.

Email: Yuhu Dai, daiyh5@mail.sysu.edu.cn; Xinsheng Peng, pengxsh@mail.sysu.edu.cn; Chuandong Lang, langchd@ustc.edu.cn

**Supplementary Figures**

**
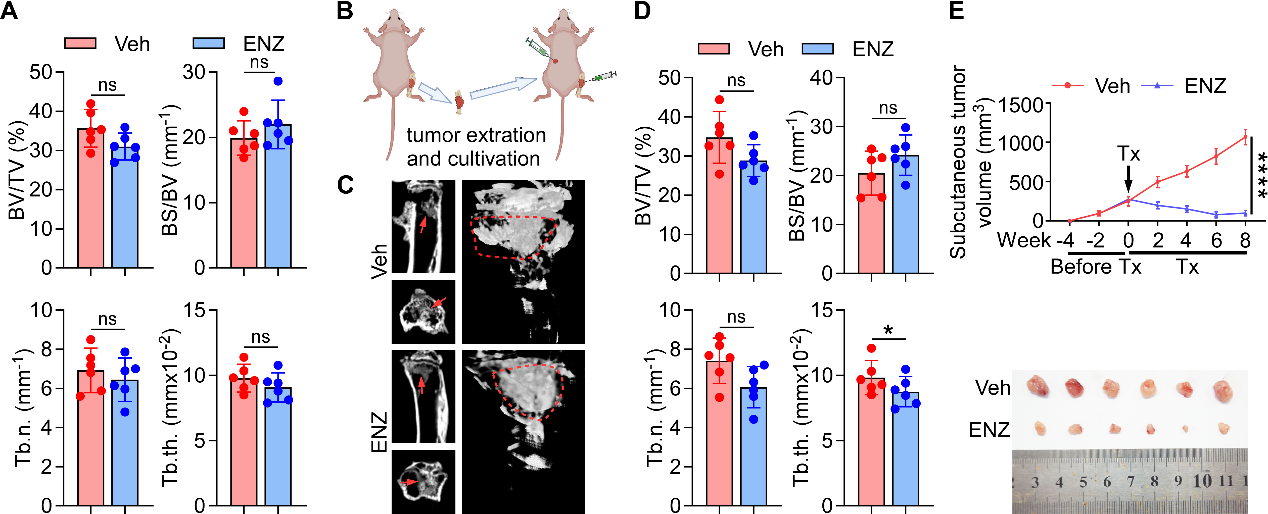
**

**Figure S1.** Osteoblasts in the bone microenvironment promote the resistance of PCa cells to ENZ. A) Quantification of bone parameters in intratibial lesions from mice after 8 weeks of treatment as indicated, including BV/TV, BS/TV, Tb.n, and Tb.th by micro-CT assay (n=6/group). B) Schematic diagram of isolating and cultivating ENZ-resistant intratibial tumor cells, and re-inoculating them into subcutaneous tissue and tibiae of new mice. C,D) Representative micro-CT images and quantification of bone parameters in intratibial lesions from mice after 8 weeks of treatment as indicated (arrows and circles indicate osteoblastic lesions, n=6/group). E) Growth curves of subcutaneous tumors in mice before and after treatment (Tx) as indicated. Representative images of subcutaneous tumors in mice after 8 weeks of treatment (n=6/group). Tumor volumes were measured weekly. ns, not significant; *, *P* < 0.05; **, *P* < 0.01; ***, *P* < 0.001; ****, *P* < 0.0001.

**
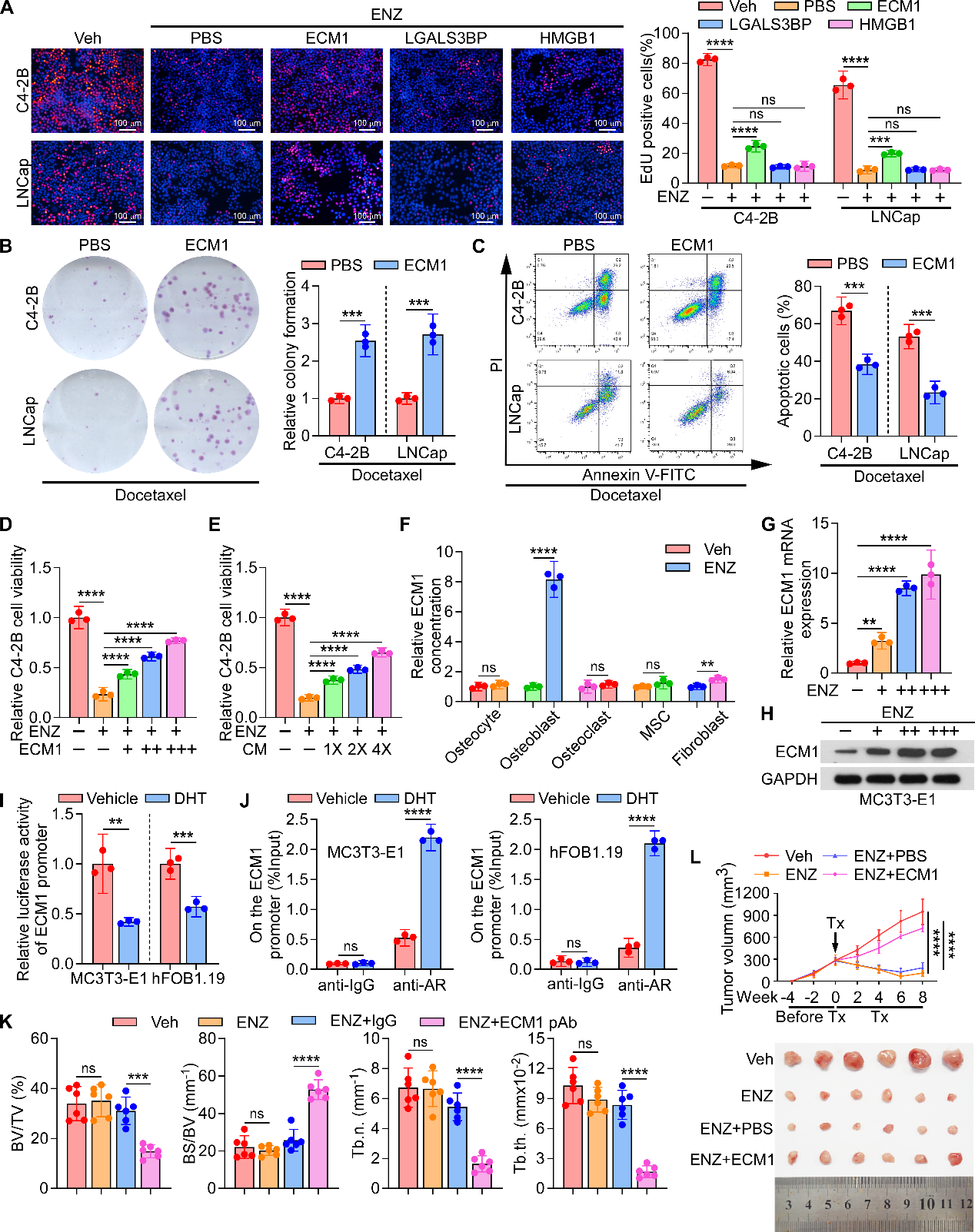
**

**Figure S2.** Osteoblast-derived ECM1 induces PCa cell resistance to ENZ. A) Representative images and quantification of cellular proliferation assessed by EdU assay in C4-2B and LNCaP cells treated with ENZ (10 μM) with the addition of either PBS, ECM1 (200 ng/mL), LGALS3BP (200 ng/mL), or HMGB1 (200 ng/mL) protein, compared to untreated cells (Scale bar, 100 µm). B) Representative images and quantification of surviving colonies formed by C4-2B and LNCaP cells treated with PBS or ECM1 protein (200 ng/mL) in the presence of docetaxel (10 μM). C) Flow cytometry analysis showing representative images and quantification of apoptosis in C4-2B and LNCaP cells treated as grouped in B. D,E) Cell proliferation on day 7 of C4-2B cells treated with increasing concentrations of ECM1 (0, 200, 400, 800 ng/mL) or CM (0, 1X, 2X, 4X) with the addition of ENZ (10 μM), compared to untreated cells. F) Quantification of ECM1 expression in the indicated cells treated with Veh (DMSO) or ENZ (10 μM) using ELISA analysis. G,H) qRT-PCR or WB analysis of ECM1 mRNA or protein expression in osteoblasts (MC3T3-E1) treated with increasing concentrations of ENZ (0, 10 μM, 20 μM, 40 μM). I) ECM1 promoter activity measured by dual-luciferase reporter assay in MC3T3-E1 and hFOB1.19 cells after vehicle (DMSO) or DHT treatment. J) ChIP-qPCR analysis of AR enrichment on the ECM1 promoter in MC3T3-E1 and hFOB1.19 cells with or without DHT treatment. K) Quantification of bone parameters in intratibial lesions from mice after 8 weeks of treatment as indicated (n=6/group). L) Growth curves of subcutaneous tumors in mice treated daily with oral Veh, ENZ (20 mg/kg), ENZ (20 mg/kg) combined with subcutaneous injection of either PBS or ECM1 protein (1 µg/2 × 10^6^ cells) twice weekly at 4 weeks before treatment, and at weeks 0, 6, and 8 during treatment (n=6/group). Representative images of subcutaneous tumors in mice after 8 weeks of treatment (n=6/group). Tumor volumes were measured weekly. ns, not significant; *, *P* < 0.05; **, *P* < 0.01; ***, *P* < 0.001; ****, *P* < 0.0001.

**
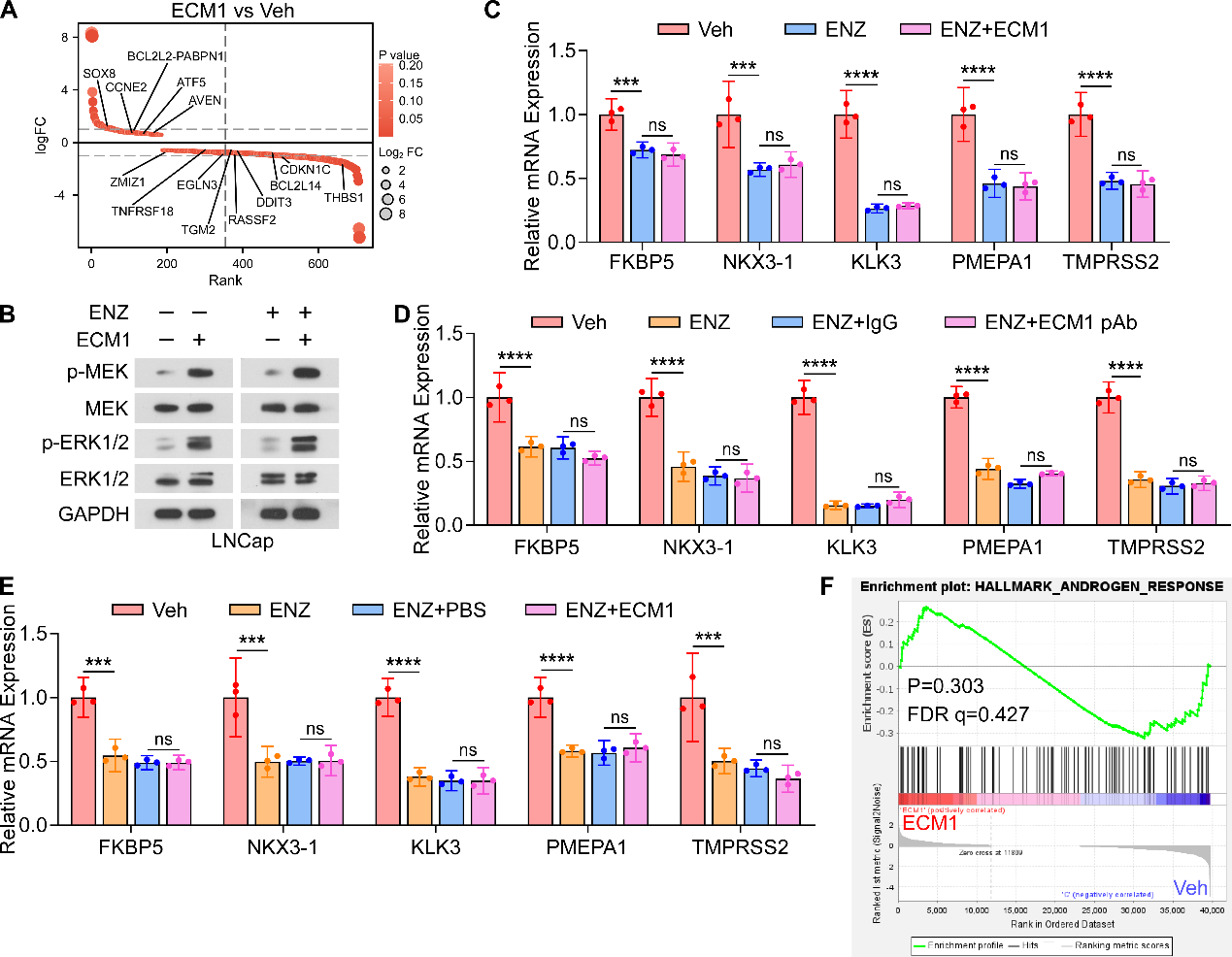
**

**Figure S3.** ECM1 activates the MAPK signaling pathway in PCa cells. A) Waterfall plot showing differentially expressed genes (p < 0.05, log2 fold change>1.5) in C4-2B cells treated with ECM1 protein (200 ng/mL, 48 h) or Veh (PBS). The highlighted genes were related to proliferation and apoptosis. B) WB analysis of MEK, p-MEK, ERK1/2, and p-ERK1/2 expression in the indicated groups of LNCap cells. C) qRT-PCR analysis of AR target genes in C4-2B cells treated as indicated, including FKBP5, NKX3-1, KLK3, PMEPA1 and TMPRSS2. D,E) qRT-PCR analysis of AR target genes in intratibial and subcutaneous tumors of mice treated as indicated. F) GSEA analysis showing the enrichment of AR signaling-related genes in the ECM1 treatment group or Veh group. ns, not significant; *, *P* < 0.05; **, *P* < 0.01; ***, *P* < 0.001; ****, *P* < 0.0001.


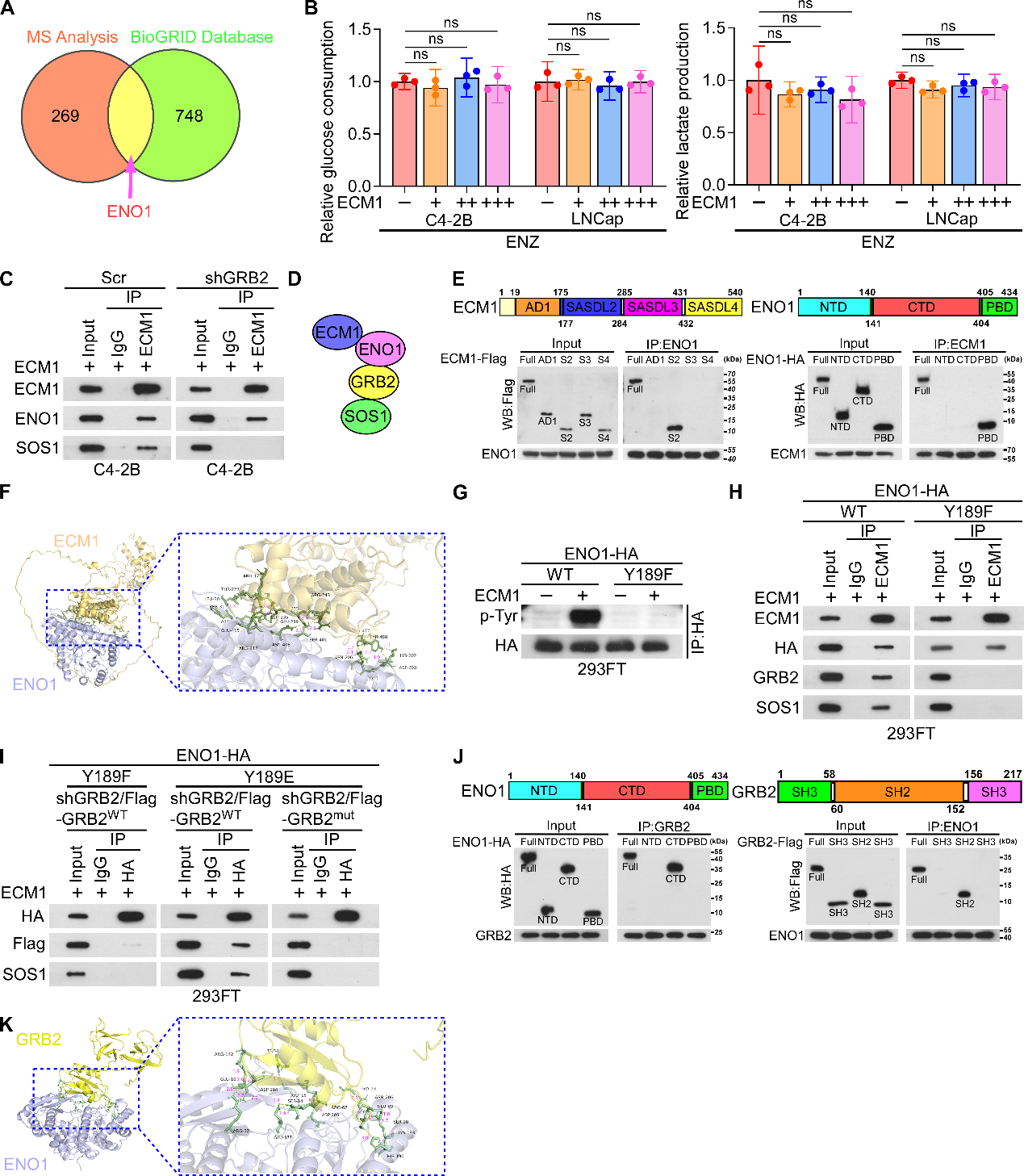


**Figure S4.** Phosphorylated ENO1 bridges ECM1 with GRB2 and SOS1 at the membrane. A) ENO1 was specifically highlighted within the intersection of proteins binding to GRB2 as determined by the BioGRID database and the ECM1 interacting proteins discovered by MS analysis. B) Quantification of glucose consumption and lactate production in C4-2B and LNCaP cells treated with ECM1 (0, 200, 400, 800 ng/mL) in the presence of ENZ (10 μM). C) IP detections of the interaction between ECM1 and ENO1 as well as SOS1 in the indicated C4-2B cells in the presence of ECM1 (200 ng/mL). D) Schematic diagram illustrating the interaction of ECM1 with GRB2 and SOS1 mediated by ENO1. E) Schematic diagram of ECM1 and ENO1 protein structures (top). Co-IP analysis using anti-ENO1 or anti-ECM1 antibodies in the indicated cells (bottom). F) 3D structure of the interaction region between ECM1 and ENO1. G) Tyr phosphorylation levels of HA-ENO1-WT and -Y189F in 293FT cells with or without ECM1 (200 ng/mL) treatment. H,I) IP assays of the interaction between ECM1 and ENO1, GRB2 as well as SOS1 in the indicated 293FT cells in the presence of ECM1 (200 ng/mL). J) Schematic diagram of ENO1 and GRB2 protein structures (top). Co-IP analysis using anti-GRB2 or anti-ENO1 antibodies in the indicated cells (bottom). K) 3D structure of the interaction region between ENO1 and GRB2. ns, not significant; *, *P* < 0.05; **, *P* < 0.01; ***, *P* < 0.001; ****, *P* < 0.0001.


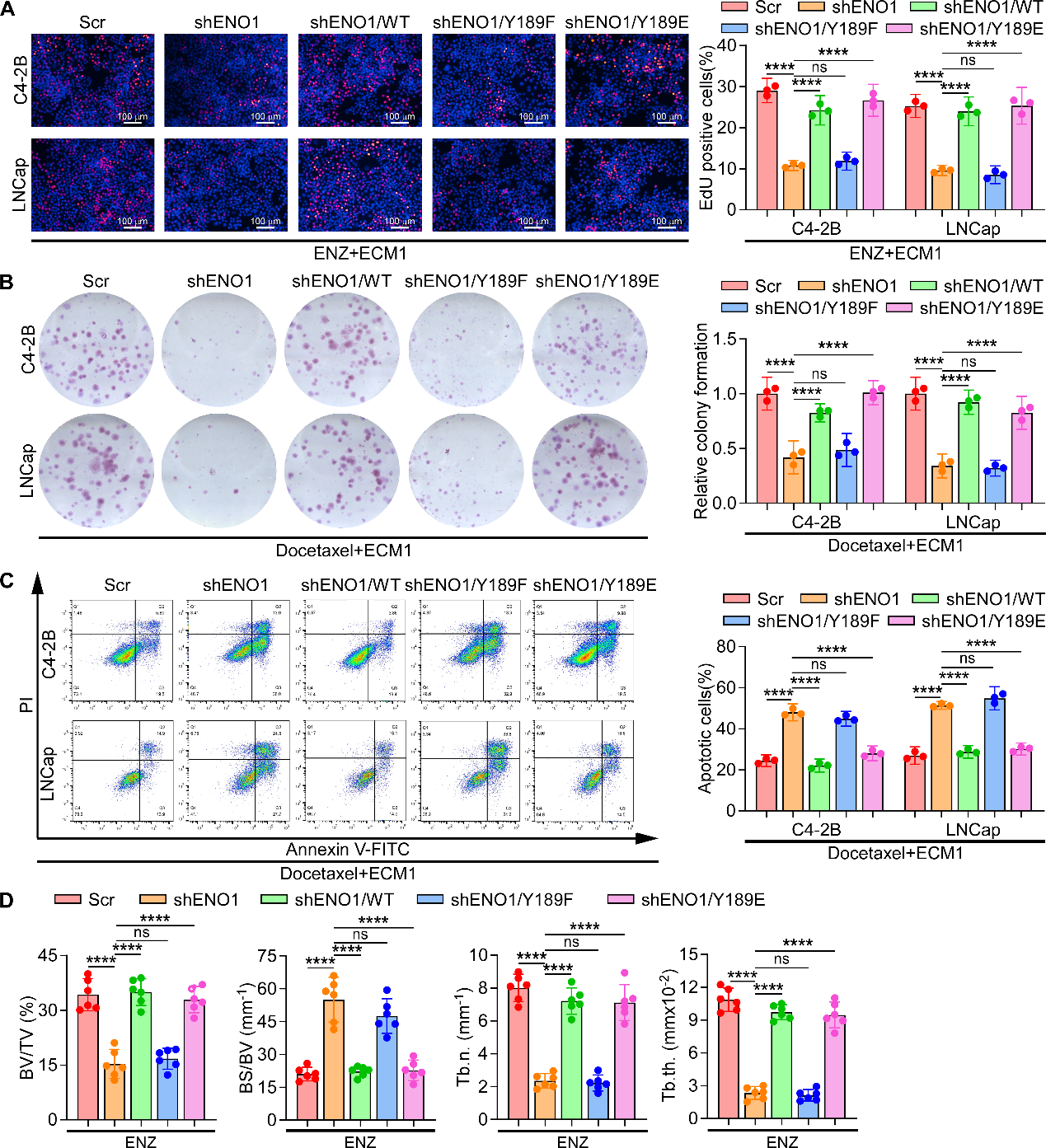


**Figure S5.** The phosphorylation of ENO1 is indispensable for ENZ resistance. A) Representative images (left) and quantification (right) of cellular proliferation assessed by EdU assay in the indicated C4-2B and LNCaP cells treated with ENZ (10 μM) with ECM1 (200 ng/mL) (Scale bar, 100 µm). B) Representative images (left) and quantification (right) of surviving colonies formed by the indicated C4-2B and LNCaP cells treated with ECM1 (200 ng/mL) and docetaxel (10 μM). C) Flow cytometry analysis showing representative images (left) and quantification (right) of apoptosis in the indicated C4-2B and LNCaP cells after ECM1 (200 ng/mL) and docetaxel (10 μM) treatment. D) Quantification of bone parameters in intratibial lesions from mice after 8 weeks of treatment as indicated (n=6/group). ns, not significant; *, *P* < 0.05; **, *P* < 0.01; ***, *P* < 0.001; ****, *P* < 0.0001.

**
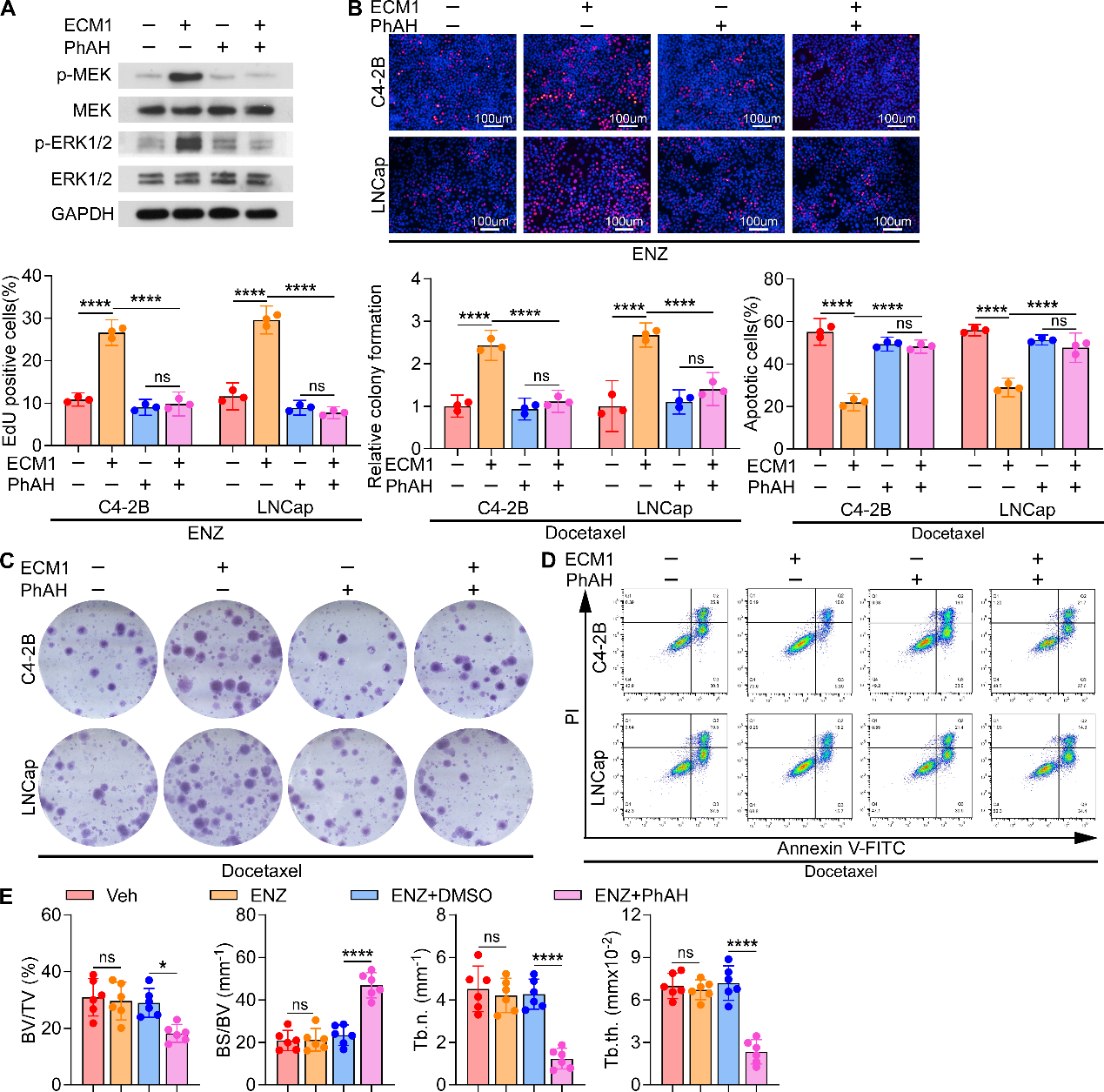
**

**Figure S6.** PhAH attenuates ENO1-mediated PCa cell resistance to ENZ. A) WB analysis of MEK, p-MEK, ERK1/2, and p-ERK1/2 expression in C4-2B cells with or without ECM1 (200 ng/mL) or PhAH (1 μM) treatment. B) Representative images (top) and quantification (bottom) of cellular proliferation assessed by EdU assay in C4-2B and LNCaP cells treated with or without ECM1 (200 ng/mL) or PhAH (1 μM) with addition of ENZ (10 μM) (Scale bar, 100 µm). C) Representative images (bottom) and quantification (top) of surviving colonies formed by C4-2B and LNCaP cells treated with or without ECM1 (200 ng/mL) or PhAH (1 μM) in the presence of docetaxel (10 μM). D) Flow cytometry analysis showing representative images (bottom) and quantification (top) of apoptosis in C4-2B and LNCaP cells treated with or without ECM1 (200 ng/mL) or PhAH (1 μM) after docetaxel (10 μM) treatment. E) Quantification of bone parameters in intratibial lesions from mice after 8 weeks of treatment as indicated (n=6/group). ns, not significant; *, *P* < 0.05; **, *P* < 0.01; ***, *P* < 0.001; ****, *P* < 0.0001.

**Supplementary tables**

**Table S1. List of primers used for qRT-PCR.**

| Gene name | Primer (5’-3’) |
| --- | --- |
| ECM1-F | AGTCCAGACCTCACACTGGT |
| ECM1-R | GCCAACTCATGAACTGGGGT |
| FKBP5-F | GCGAAGGAGAAGACCACGACAT |
| FKBP5-R | TAGGCTTCCCTGCCTCTCCAAA |
| NKX3-1-F | CGCAGAACGACCAGCTGAGCA |
| NKX3-1-R | CCTGAAGTGTTTTCAGAGTCCAAC |
| KLK3-F | CGCAAGTTCACCCTCAGAAGGT |
| KLK3-R | GACGTGATACCTTGAAGCACACC |
| PMEPA1-F | CTGAGCCACTACAAGCTGTCTG |
| PMEPA1-R | GGATTCCGTTGCCTGACACTGT |
| TMPRSS2-F | CCTCTAACTGGTGTGATGGCGT |
| TMPRSS2-R | TGCCAGGACTTCCTCTGAGATG |
| GAPDH-F | GTCTCCTCTGACTTCAACAGCG |
| GAPDH-R | ACCACCCTGTTGCTGTAGCCAA |

ECM1: Extracellular Matrix Protein 1;

FKBP5: FKBP Prolyl Isomerase 5;

NKX3-1: NK3 Homeobox 1;

KLK3: Kallikrein Related Peptidase 3;

PMEPA1: Prostate Transmembrane Protein, Androgen Induced 1;

TMPRSS2: Transmembrane Serine Protease 2;

GAPDH: Glyceraldehyde-3-Phosphate Dehydrogenase.
